# Pangenome and genomic signatures linked to the dominance of the lineage-4 of *Mycobacterium tuberculosis* isolated from extrapulmonary tuberculosis patients in western Ethiopia

**DOI:** 10.1371/journal.pone.0304060

**Published:** 2024-07-25

**Authors:** Basha Chekesa, Harinder Singh, Norberto Gonzalez-Juarbe, Sanjay Vashee, Rosana Wiscovitch-Russo, Christopher L. Dupont, Musse Girma, Oudessa Kerro, Balako Gumi, Gobena Ameni

**Affiliations:** 1 Aklilu Lemma Institute of Pathobiology, Addis Ababa University, Addis Ababa, Ethiopia; 2 Collage of Natural and Computational Science, Wallaga University, Nekemte, Ethiopia; 3 J. Craig Venter Institute, Rockville, Maryland, United States of America; 4 J. Craig Venter Institute, La Jolla, California, United States of America; 5 Institute of Agriculture, The University of Tennessee, Tennessee, Knoxville, United States of America; 6 College of Agriculture and Veterinary Medicine, United Arab Emirates University, Al Ain, United Arab Emirates; The University of Georgia, UNITED STATES

## Abstract

**Background:**

The lineage 4 (L4) of *Mycobacterium tuberculosis* (MTB) is not only globally prevalent but also locally dominant, surpassing other lineages, with lineage 2 (L2) following in prevalence. Despite its widespread occurrence, factors influencing the expansion of L4 and its sub-lineages remain poorly understood both at local and global levels. Therefore, this study aimed to conduct a pan-genome and identify genomic signatures linked to the elevated prevalence of L4 sublineages among extrapulmonary TB (EPTB) patients in western Ethiopia.

**Methods:**

A cross-sectional study was conducted at an institutional level involving confirmed cases of extrapulmonary tuberculosis (EPTB) patients from August 5, 2018, to December 30, 2019. A total of 75 MTB genomes, classified under lineage 4 (L4), were used for conducting pan-genome and genome-wide association study (GWAS) analyses. After a quality check, variants were identified using MTBseq, and genomes were *de novo* assembled using SPAdes. Gene prediction and annotation were performed using Prokka. The pan-genome was constructed using GET_HOMOLOGUES, and its functional analysis was carried out with the Bacterial Pan-Genome Analysis tool (BPGA). For GWAS analysis, Scoary was employed with Benjamini-Hochberg correction, with a significance threshold set at p-value ≤ 0.05.

**Results:**

The analysis revealed a total of 3,270 core genes, predominantly associated with orthologous groups (COG) functions, notably in the categories of ‘[R] General function prediction only’ and ‘[I] Lipid transport and metabolism’. Conversely, functions related to ‘[N] Cell motility’ and ‘[Q] Secondary metabolites biosynthesis, transport, and catabolism’ were primarily linked to unique and accessory genes. The pan-genome of MTB L4 was found to be open. Furthermore, the GWAS study identified genomic signatures linked to the prevalence of sublineages L4.6.3 and L4.2.2.2.

**Conclusions:**

Apart from host and environmental factors, the sublineage of L4 employs distinct virulence factors for successful dissemination in western Ethiopia. Given that the functions of these newly identified genes are not well understood, it is advisable to experimentally validate their roles, particularly in the successful transmission of specific L4 sublineages over others.

## Introduction

Tuberculosis (TB) continues to pose a substantial global public health challenge. In 2022, approximately 10.6 million people worldwide fell ill with TB, and among them, 1.30 million succumbed to the disease, including individuals with HIV [1]. Pulmonary TB (PTB) is the most common clinical manifestation, but a considerable number of patients develop extrapulmonary TB (EPTB), affecting various organs or tissues, including aggressive presentations such as lymph nodes and central nervous system TB [2]. EPTB constitutes about 15% of reported TB cases worldwide and exceeds 30% of TB cases in Ethiopia [3]. Despite being a debilitating disease, EPTB often faces neglect as a public health concern, particularly in developing countries like Ethiopia.

TB is caused by members of the *Mycobacterium tuberculosis* complex (MTBC), which is classified into nine phylogenetic lineages [4]: L1 (Indo-Oceanic), L2 (East Asian), L3 (East African-Indian), L4 (Euro-American), L5 (*M*. *africanum* West-African 1), L6 (*M*. *africanum* West-African 2), L7 (Ethiopia), L8 (MTB from the African Great Lakes), and the recently described *M*. *africanum* L9. These MTBC lineages exhibit variations in virulence, geographic distribution, and prevalence across different regions globally. L4 stands out as the most widely distributed lineage globally [5], yet the factors contributing to its global prevalence remain unknown.

Variations between MTBC lineages extend to the sub-lineage level, influenced by factors such as host genetics and environmental variables, including antibiotic resistance. Increased transmission of drug-resistant TB compared to drug-susceptible strains could contribute to these variations [6]. However, the dominance of certain lineages, even in the absence of drug resistance [7], suggests that genetic factors within the bacteria also play a role. The genetic determinants contributing to the success of specific MTBC lineages in transmitting TB, particularly in the context of EPTB, remain unclear. Further studies are essential to identify these genetic determinants.

In Ethiopia, L4 is one of the most prevalent lineages, with its sub-lineages, specifically L4.2 (T3-ETH/SIT149) and L4.6 (T3/SIT37), showing high circulation in the country [8, 9]. Previous unpublished research in western Ethiopia, conducted by our team, revealed that L4.6.3 and L4.2.2.2 were the predominant sublineages. Despite this, the genetic factors within L4 strains, contributing to their overall success and that of their sublineages, remain unexplored to date.

Comparative genomic analysis is a potent tool enabling the comparison of multiple strains across various categories [10]. Whole genome sequencing (WGS) of bacteria is commonly performed in numerous laboratories worldwide, although it is relatively uncommon in Ethiopia. This process generates vast quantities of precise genome data, the majority of which remains poorly understood [11]. Establishing a pan-genome is crucial, as it significantly aids in gene discovery (utilized as a drug target and vaccine candidate) and enhances comprehension of the genome architecture of a species. The pan-genome represents the genomic repertoire of a species, addressing questions related to diverse phenotypes exhibited by individuals of that species [12]. This pan-genome study of L4 of MTB aims to delineate a conserved core genome and a dynamic accessory genome of MTB.

Moreover, the advent of large-scale bacterial GWAS has facilitated the identification of genes or genomic variants linked to outbreaks, evolution, antibiotic resistance, pathogenicity, transmission, and host-adaptive traits [13]. Despite the pivotal role of GWAS in genomics, the application of bacterial GWAS is a relatively recent development, and as of now, no such study has been conducted on MTB in Ethiopia. Hence, the objective of this study was to evaluate the pan-genome of 75 clinical isolates of L4 MTB and to conduct a GWAS to identify novel genes and their genomic variants associated with the high prevalent (L4.6.3 and L4.2.2.2) or low prevalence L4 sublineages of MTB.

## Materials and methods

### Study setting

The research was carried out at Nekemte Specialized Hospital and Wallaga University Referral Hospital in Nekemte City, the capital of East Wallaga Zone, located approximately 320km west of Addis Ababa. These hospitals were selected as they serve as the primary EPTB diagnosis and treatment centers in western Ethiopia. An institutional-based, cross-sectional study was conducted on confirmed EPTB patients who visited the two hospitals. The study included 75 participants from all age groups, while those unwilling to provide consent were excluded from the study.

### Collecting, transporting, and culturing specimens

Following the participants’ consent, fine needle aspiration (FNA) specimens were collected between August 5, 2018, and December 30, 2019. These specimens were then transported in a packed ice box at +4°C to the TB laboratory at Aklilu Lemma Institute of Pathobiology (ALIPB), Addis Ababa University (AAU), for screening for the growth of MTBC isolates. The culturing of samples was conducted using the Petroff procedure at ALIPB, AAU [14]. Out of the 264 specimens collected, 121 tested positive in culture, and among them, 96 isolates were chosen randomly for sequencing due to resource constraints, as sequencing all 121 isolates was not feasible. From the sequenced samples, we specifically utilized subsets of 75 samples belonging to L4. Samples from L3 and L7, as well as reads with a mean coverage of < x20 and genome assemblies with a contamination level of > 5%, were excluded from the analysis.

### DNA extraction and whole-genome sequencing

DNA extraction was conducted using a modified chloroform and acetyl trimethyl ammonium bromide (CTAB) protocol, as previously described [15]. Subsequently, the DNA was sent to the J. Craig Venter Institute (JCVI), USA for pan-genome and gene signature studies. DNA concentration was quantified using Qubit-4 technology and a Qubit dsDNA HS Assay kit (Thermo Fisher Scientific, Waltham, USA). A genomic DNA concentration of 1ng was employed to prepare sequencing libraries with the Illumina Nextera XT library preparation kit (Illumina, San Diego, USA) following the manufacturer’s instructions. Quality control was performed using the Agilent High Sensitivity DNA kit (Agilent, CA, USA) and Qubit dsDNA HS Assay kit (Thermo Fisher Scientific, Waltham, USA). The libraries were manually normalized based on DNA concentration and average fragment size. WGS was carried out on the Illumina NovaSeq 6000 technology at JCVI lab using 2 × 150 paired-end chemistry, producing paired-end FastQ files [16].

### Bioinformatic analysis

#### Quality check, variant calling, and *de novo* assembly

The reads’ quality was assessed using FastQC v0.12.1 [17] both before and after trimming for the removal of adapter sequences, low-quality reads, and filtering for a minimum read length. Trimmomatic v0.39 [18] was employed for trimming with the following parameters: phred33, LEADING:3 TRAILING:3 SLIDINGWINDOW:4:15 MINLEN:36. Following a quality check, we utilized the MTBseq pipeline (version 1.0.4) [19] for the analysis of MTBC isolates. This semi-automated bioinformatics pipeline was employed to call variants, encompassing both indels and SNPs. The analysis included a minimum coverage criterion of 10 forward and 10 reverse reads, with a 75% allele frequency. Furthermore, a minimum of four read calls, each with a Phred score of at least 20, was required. Variants within a 12bp window in the same isolate and sites with ambiguous calls in over 5% of isolates were automatically excluded.

The identification of MTB species, lineages, sub-lineages, and drug-resistance profiles was conducted using TB-Profiler v5.0.1 [20] and MTBseq [19], along with mykrobe (v0.12.1) [21], based on the sequence reads. Furthermore, *de novo* assembly of MTB genomes was conducted with SPAdes v3.15.5 [22], employing different odd k-mer sizes in the range k = 21 to k = 87. Assembly statistics, such as N50, largest contig, GC-content, and genome fraction covered, were computed using Quast v5.2.0 [23] to assess the quality and compare it to the reference genome H37Rv (NC_000962.3). A working draft genome was constructed using CONTIGutor v2.7.4 [24], utilizing the complete genome of MTB strain H37Rv (GenBank accession number NC_000962.3) as a reference genome.

#### Estimating completeness and annotation

The checkM v1.2.2 [25] strategy was employed to estimate the percentage completeness, contamination level, and strain heterogeneity of the draft genome assemblies. Genomes meeting criteria of greater than 97% completeness and less than 2% contamination level were selected for subsequent analysis. For gene detection and genome annotation, the Prokka v1.14.6 autoannotation package was used with an E-value of 1e-9 and 80% coverage [26].

#### Pan-genome construction, core, and accessory genome evolution

The GET_HOMOLOGUES V09062017 tool [27] was employed to cluster homologous genes, utilizing BLASTP with a sequence identity of 90% and a default query coverage of at least 75% for paired alignments. The resulting syntenic clusters were utilized to create a pan-genome matrix, illustrating the presence and absence variants (PAVs) through *compare_clusters*.*pl*. The pan-genome matrix was then employed to categorize genes into core, soft-core, shell, and cloud genes with *parse_pangenome_matrix*.*pl*, an auxiliary script of *get_homologues*.*pl*. Core genes, a subset of soft-core, were defined as those present in all 75 genomes, while accessory genes were found in a subset of the 75 strains. The accessory gene cluster was further divided into shell and cloud gene clusters, where soft-core genes were present in 95% of the genomes. Cloud genes were present in 2 or fewer genomes, and shell genes comprised the remaining genes (70–3 isolates). The distribution of cluster sizes, based on the number of genomes these clusters contained, was visualized using R with *parse_pangenome_matrix*.*pl*. To assess the openness/closeness of the pan-genome, a theoretical estimation of the pan-genome size was conducted using an exponential model, fitting it to the OMCL accessory gene clusters. The exponential decay and growth curves for core and pangenome, respectively, were plotted using the *plot_pancore_matrix*.*pl* script provided by GET_HOMOLOGUES with the Tettelin parameters [27].

#### Functional pan-genome analysis and genome-wide association study of lineage-4 of MTB

The core genome, dispensable genome, and unique genes constituting the pan-genome were scrutinized for their functions using the Bacterial Pan-Genome Analysis Tool (BPGA) v1.3 software [28]. COG IDs were assigned to all representative protein sequences from each orthologous protein cluster by utilizing the *ublast* function of *USEARCH* against the COG (Clusters of Orthologous Groups of proteins) reference databases, with a 95% similarity threshold for orthologous clustering. Subsequently, the percentage frequencies of COG categories were computed for core genes, accessory genes, and singletons (strain-specific genes). Histogram outputs were generated using *gnuplot* to visualize the distribution of COG categories.

Association analysis was conducted using Scoary v1.6.16 [11], a Python program, to assess relationships among genes in the pan-genome matrix and prevalence phenotyping data. The pan-genome matrix, detailing PAVs, was utilized along with a matrix indicating high and low prevalence traits. Correlations between observed presence/absence and prevalence were determined using Fischer’s exact test, with genes displaying a naïve p-value < 0.05 considered potentially significant. To mitigate false positives and minimize the family-wise error rate (FWER), p-values were adjusted using Bonferroni’s (p-value < 1) and Benjamini–Hochberg (p-value < 0.05) corrections, with adjusted values indicating significance.

To validate variants (SNPs and indels) in genes significantly linked to high or low MTB strain prevalence, a confirmation process was conducted using multiple genome alignment through Mauve v2.4.0 [29] and Parsnp (part of the Harvest package, v1.1.2) [30], using H37Rv as a reference genome. Additionally, the Protter server v1.0 was employed to corroborate the structure and cellular localization of truncated genes associated with high or low prevalent MTB L4 [31].

#### Phylogenetic analysis of the MTB lineage-4

The phylogenomic reconstruction of the 75 genomes was carried out using the PAV data obtained from the consensus of ortholog groups with the COG and Ortho Markov Clustering (OMCL) algorithms in GET_HOMOLOGUES [27]. Phylogenetic trees were constructed using the parsimony method from discrete characters (present/absence of genes) through the Dnapars v3.69 software, which is part of the PHYLIP suite [32]. The resulting trees were visualized using iTOL [33].

Whole genome SNPs (wgSNPs) were extracted from the assemblies using kSNP v4.0 [34]. This program identifies high-confidence SNPs by comparing unique nucleotide sequences (k-mers) from each genome that differ only at the middle site. A k-mer size of 21 bp was utilized, and the wgSNPs alignments from our studied genomes were employed to reconstruct a maximum likelihood (ML) phylogenetic tree using RAxML [35]. The tree was built with a general time reversible (GTR) nucleotide substitution model, with 100 bootstrap estimates, and then visualized using iTOL [33].

A database containing annotated genes present in the finished genome of the MTB strain H37Rv (GenBank accession number NC_000962.3) was constructed using py MLST V2.1.4 [36]. Annotated genes from the query genomes (our 75 isolates) were then compared with the reference genome to establish a list of core genome genes (3,961). A multi-fasta alignment file generated from the script was utilized to construct a maximum likelihood tree by IQ-TREE v2.2.2.6 [37]. The maximum likelihood phylogeny was built using a TVM+F+I+I+R10 evolutionary model with ultrafast bootstrap support of 1000 replicates (–bb 1000 -alrt 1000) [37]. The IQ-tree software searches through several evolutionary models and selects for a “best-fit model” using the Bayesian Information Criterion (BIC) score assigned to each tested model. The iTol web-based phylogeny tool v6 was employed to visualize the tree [33].

### Statistical analysis

Descriptive statistics were employed to present the results. Fisher’s exact test, conducted using R, was applied to the query pan-genome data, with a statistical significance threshold set at p < 0.05.

### Ethical clearance

Ethical approval for this study was secured from the Addis Ababa University, Aklilu Lemma Institute of Pathobiology Institutional Review Board (ALIPB/IRB/011/2017/2018). Study participants were recruited from August 5, 2018, to December 30, 2019. Written informed consent and assent were obtained from each participant before the collection of fine needle aspiration (FNA) samples. Additionally, for participants below the age of 16, informed consent was obtained from the parents of the study participants. The study was conducted in compliance with applicable guidelines and regulations.

## Results

### Prevalence and genomic features of the L4 of MTB

In a prior unpublished study, the results revealed that L4 was the predominant lineage, comprising 87.64% of the total 89 MTB isolates. Among these, 78 were classified as L4 (of which 75 isolates were used in this study), 10 as L3, and 1 as L7, all originating from EPTB cases in western Ethiopia. In the L4 category, sublineages L4.6.3 and L4.2.2.2 were the most prevalent, constituting the highly prevalent group, with proportions of 34.67% (26/75) and 26.67% (20/75) of the L4 isolates, respectively. Conversely, the remaining 29 L4 sub-lineages were classified as low prevalent groups (S1 Fig and S1 Table). The study included 38 female and 34 male participants, with a mean age of 29 years (ranging from 2 to 65 years) (S1 Table). Detailed genomic features of the 75 isolates are presented in S1 and S2 Tables, and S2 Fig.

### Phylogenetic analysis of the MTB L4

**Fig 1:**
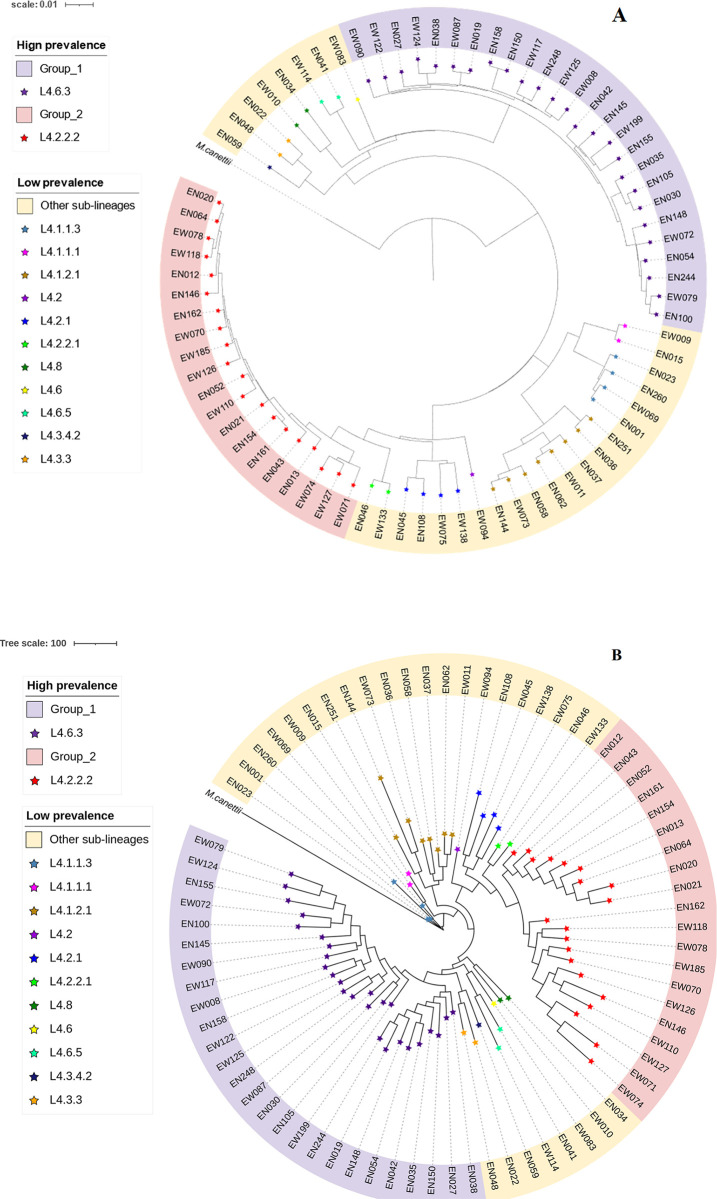
Phylogenetic analysis of MTB L4 strains from EPTB phenotypes of the diseases. (**A**) Whole genome SNPs based phylogeny by maximum likelihood, and (**B**) Presence and absence variations based parsimonious phylogeny. The outgroup was *M*. *canetti* CIPT 140010059 and the shapes on each tip of all branches indicate sub-lineages of L4. The red shade corresponds to isolates with a high prevalence of L4.2.2.2 sub-lineage, the light purple corresponds high prevalence of L4.6.3 and the yellow shades correspond to all isolates with a low prevalence in western Ethiopia.

A phylogenetic tree was reconstructed from 22,380 wgSNPs present in the genomes of 75 L4 MTB isolates (Fig 1A). Additionally, PAV identified using GET_HOMOLOGUES allowed for the phylogenomic reconstruction of L4 MTB isolates, capturing the phylogeny implicit in the matrix (Fig 1B). We also employed a wgMLST-based approach to construct a phylogenomic tree using 168,977 alleles distributed in the annotated coding sequences of 75 L4 MTB sub-lineages (S3 Fig). The *M*. *canetti* CIPT 140010059 strain was used as an outgroup.

The PAVs-based phylogeny, when compared to wgSNPs and wgMLST-based phylogenies, accurately distinguished the high-prevalence L4.6.3 and L4.2.2.2 strains from the low-prevalence group, which included various sub-lineages (S1 and S2 Figs). All three approaches aligned with the classification of sub-lineages reported by Spoligotyping and WGS of the TB-profiler. This further supports that PAVs could offer a phylogenomic resolution of highly prevalent and low-prevalence MTB L4 strains, similar to that of wgSNPs and wgMLST.

### Pan-genome and core-genome L4 of MTB

**Fig 2:**
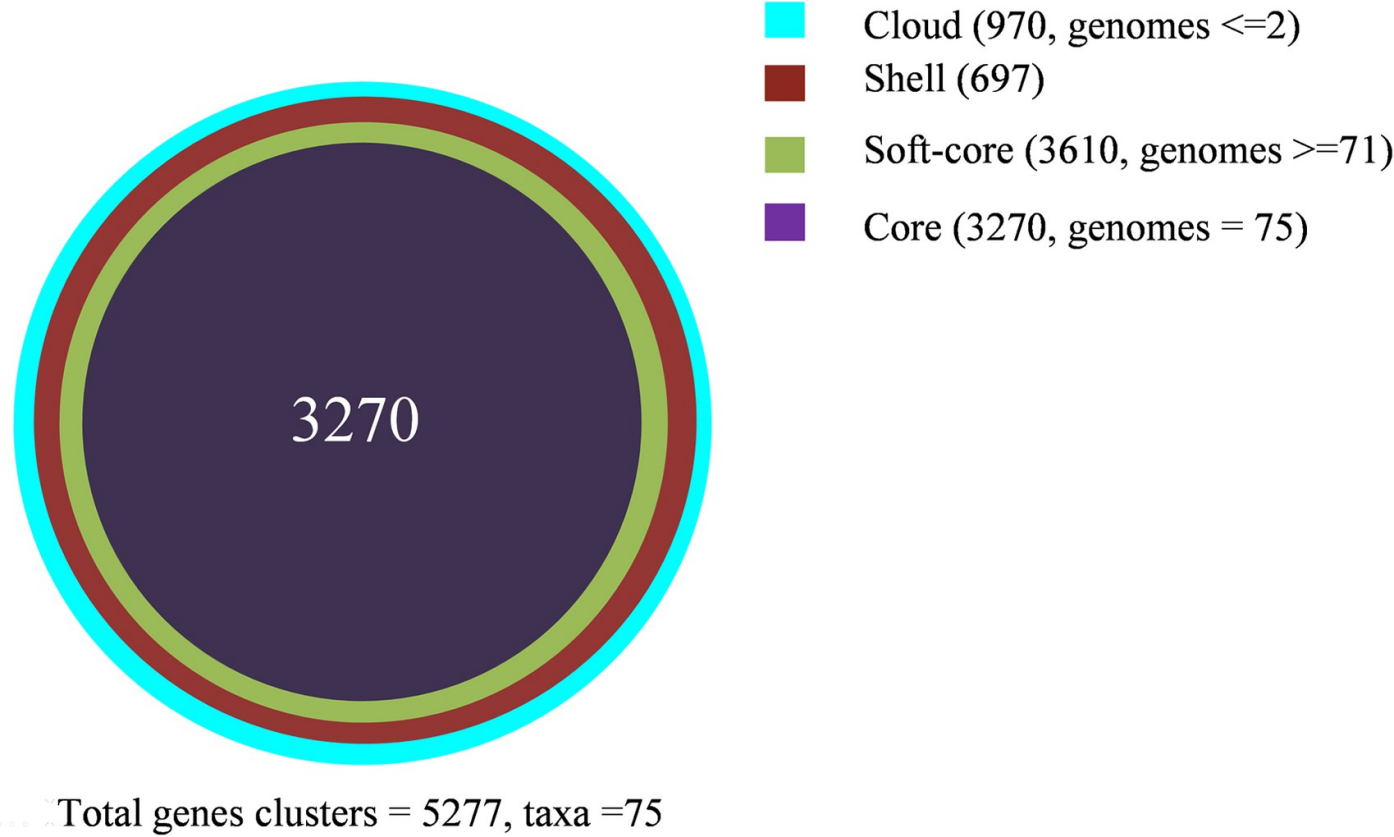
Pan-genomic area of L4 of MTB. The global composition of gene clusters is divided into four compartments. Different clusters of the genes are marked with different colors.

In this study, the combination of the Cluster of Orthologous Sequences (COG) and OMCL algorithms yielded a total of 5,277 gene clusters (S4 Fig). Furthermore, the intersection of COG, Bidirectional Best Hit (BDBH), and OMCL algorithms were utilized to identify the core genes (S5 Fig). The complete set of 5,277 gene clusters from the pan-genome was classified into four occupancy classes: core, soft-core, shell, and cloud (Fig 2). The core compartment had the highest occupancy, accounting for 3,270 gene clusters (61.97%), while the shell compartment had the lowest, with 697 gene clusters (13.21%). Additionally, cloud clusters represented 18.38% of the pan-genome (Fig 2).

**Fig 3:**
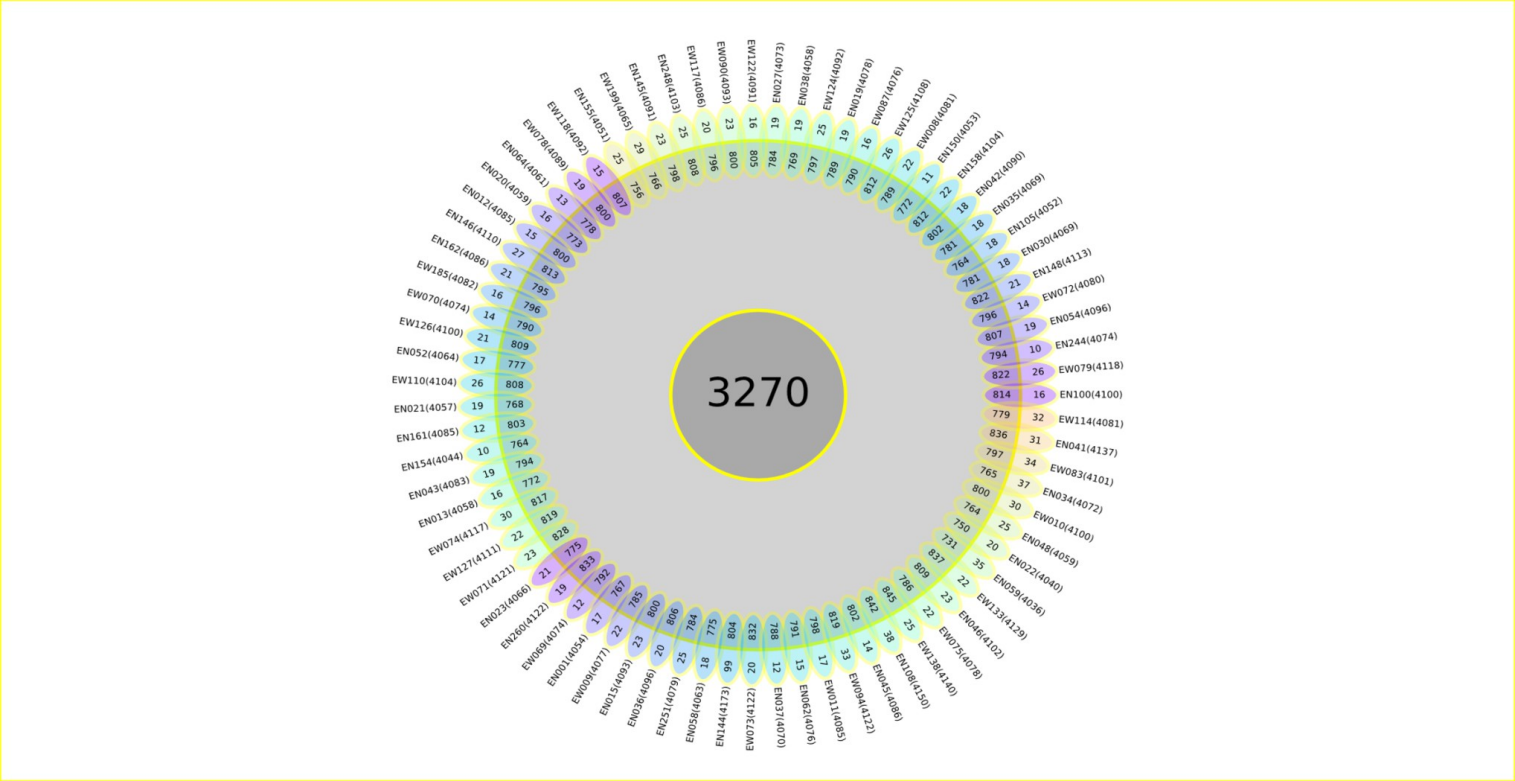
Flower plot showing the core, accessory, and strain-specific genes of the 75 L4 MTB isolates. It shows all L 4 of MTB isolates that make up the pan-genome. The flower plot shows the core gene number (in the center), the second clear circle shows the accessory genes and the petals show the number of specific genes of each isolate in the 75 genomes. The numbers below each isolate denote the total number of related CDSs.

The accessory genome exhibited an average of 795.05 ± 22.28 genes (mean ± standard deviation), including 22 ± 10.98 strain-specific genes. Each isolate, on average, had 4,087.05 ± 26.08 annotated coding sequences (CDS) (Fig 3).

### Open pan-genome of L 4 of MTB

**Fig 4:**
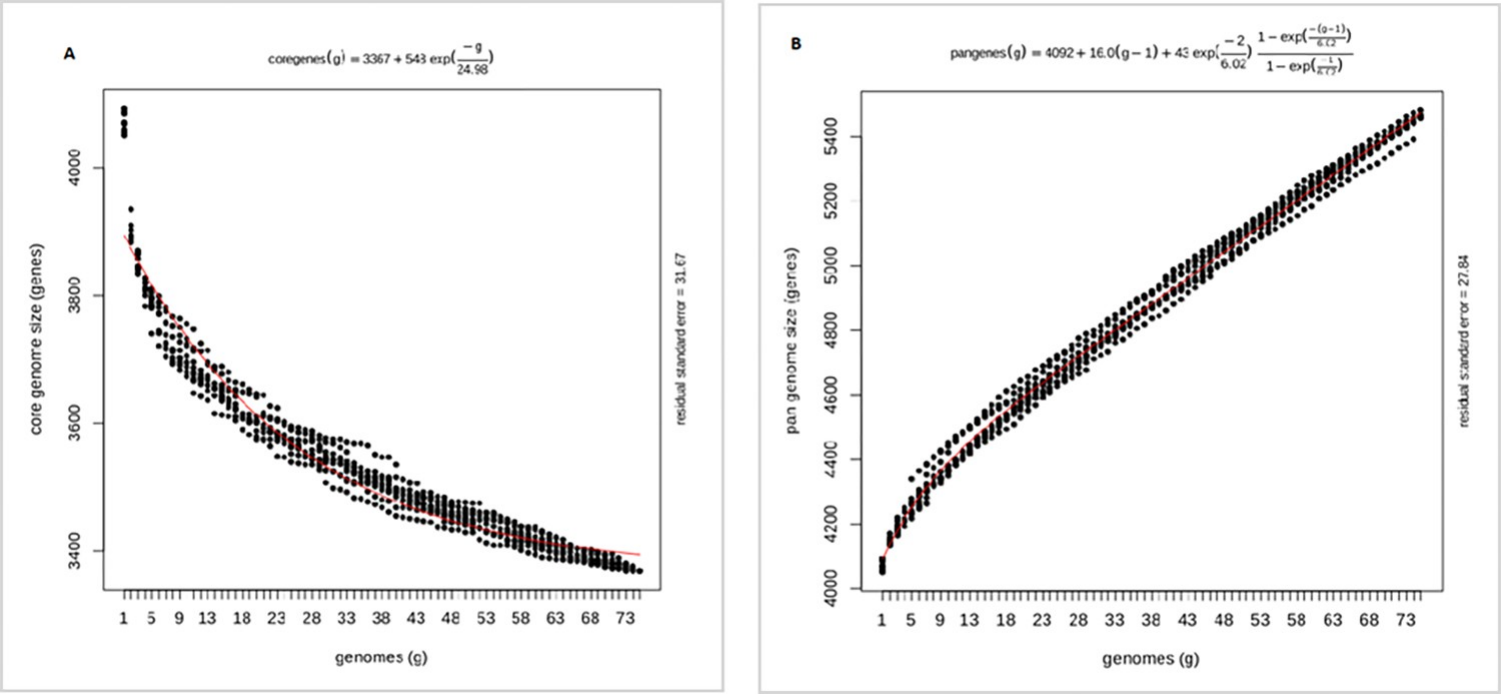
Theoretical estimation of the core and pan-genome sizes based on the exponential model. (**A**) Estimation of core genome size based on Tettelin exponential decay model fit to OMCL clusters. A pan-genome is considered almost closed when the curve representing the total number of genes (pan-genome curve) ceases to significantly increase with the addition of new genomes (**B**) Estimation of pan-genome size based on Tettelin exponential growth model fit OMCL clusters.

The exponential decay model, based on the OMCL algorithm, predicted a theoretical core genome of 3,367 genes, indicating that the number of orthologous gene clusters in the core tends to asymptote near this value (Fig 4A). In contrast, the exponential growth model suggested that the pan-genome continues to grow linearly above 5,400 gene clusters, indicating an open pan-genome for L4 of MTB. This suggests that the pan-genome is continuously acquiring new genes, and adapting to different environments, with an approximate increase of 16 genes each time a new genome is added (Fig 4B).

### Functional pan-genome annotation

**Fig 5:**
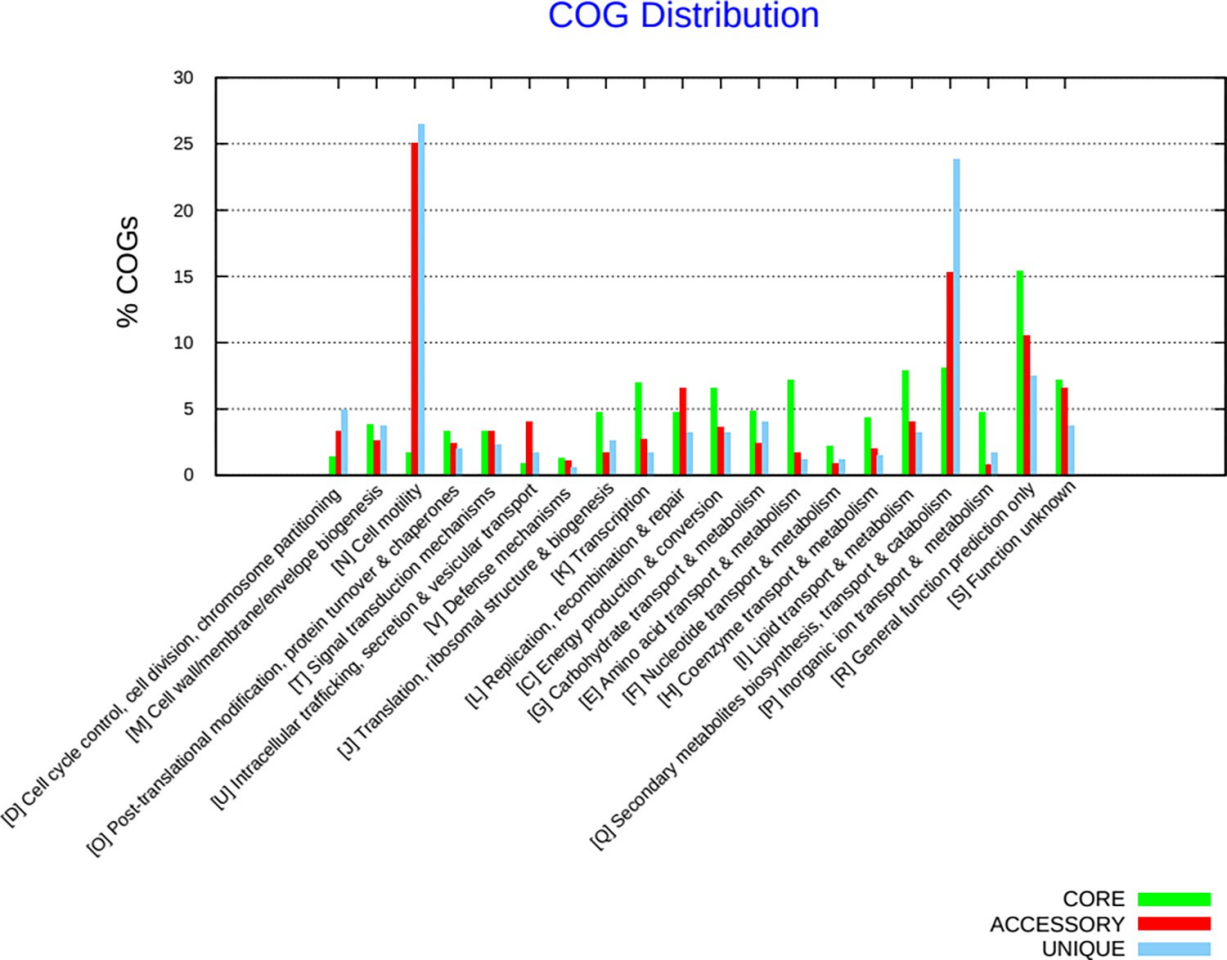
Functional classes of L4 of MTB core genes (green), accessory (red), and unique (light blue) across COG categories. The COG functional categories are shown on the x-axis. The percentage of related genes for each COG functional category is shown on the y-axis.

The predicted functions of coding sequences (CDS) inferred from annotated protein sequences in the COG (Clusters of Orthologous Groups) database for MTB strains revealed the top five enriched COG categories, along with their respective percentages: [R] General function prediction only (15.34%), [I] Lipid transport and metabolism (7.88%), [S] Function unknown (7.17%), [E] Amino acid transport and metabolism (7.14%), and [K] Transcription (6.94%). Notably, these categories showed a higher proportion of core genes compared to accessory and unique genes. Additionally, the analysis of COG distribution highlighted that functions related to ’[N] cell motility’ and ’[Q] secondary metabolites biosynthesis, transport, and catabolism’ exhibited a higher overall proportion in unique and accessory genes as opposed to core genes, as depicted in Fig 5.

### GWAS of L4 of MTB

In the association study aiming to identify genes responsible for the high prevalence of the L4.6.3 sublineage of MTB, a correlation was established between the prevalence phenotype and the presence or absence of genes in the pan-genome. The Benjamini-Hochberg correction was applied due to the conservative nature of Bonferroni’s multi-testing correction, with a significance threshold of p-value ≤ 0.05. When comparing the high-prevalence L4.6.3 and low-prevalence groups, 11 genes exhibited significant associations. Among these, five genes were annotated, while the remaining six were non-annotated hypothetical protein-coding genes (Table 1). Specifically, four genes (*Rv1928c*, *Rv3093c*, *Rv0025*, and *PE_PGRS20*) showed significant associations with the highly prevalent L4.6.3 sublineage, while the other seven genes (*Rv0071*, *vapC28*, *Rv3098A*, *icl2*, *fadD34*, *PE_PGRS6*, and *Rv2994*) were significantly associated with the less prevalent phenotype (Table 1).

**Table 1:** MTB L4’s genes associated with L4.6.3 or low prevalent sublineages identified in GWAS analysis.

Gene	Annotation	Uniprot code	Length (aa)	Genomes number high prev. N = 26	Genomes number low prev. N = 29	P-value	Bonferroni’s-adjusted P-value^a^	Benjamini-H. adjusted P-value^a^	Gene association
***Rv0071***	Maturase	O53616	235	0	29	2.81E-16	5.05E-13	7.22E-14	LP
***vapC28/Rv0609***	Ribonuclease	P9WF81	133	0	29	2.81E-16	5.05E-13	7.22E-14	LP
***Rv3098A***	PemK-like protein	V5QRX7	106	0	29	2.81E-16	5.05E-13	7.22E-14	LP
***icl2/aceA***	Isocitrate lyase 2	Q8VJU4	766	0	24	5.18E-09	9.31E-06	3.58E-07	LP
***Rv1928c***	Short-chain type dehydrogenase/reductase	P95286	255	26	8	5.18E-09	9.31E-06	3.58E-07	HP
***PE_PGRS20***	PE-PGRS family protein	P9WIF9	463	26	11	2.51E-07	0.0005	1.46E-05	HP
***Rv3093c***	Oxidoreductase	O05772	334	26	13	2.46E-06	0.004	0.0001	HP
***fadD34***	Fatty-acid—CoA ligase	L7N699	562	13	29	7.17E-06	0.01	0.0003	LP
***PE_PGRS6***	PE-PGRS family protein	L0T3X8	594	13	29	7.17E-06	0.01	0.0003	LP
***Rv0025***	Hypothetical protein	P9WMA1	120	26	15	2.00E-05	0.05	0.0007	HP
***Rv2994***	MFS-type transporter	P9WJW7	445	16	29	0.0002	0.32	0.005	LP

*HP* high prevalence, *LP* low prevalence.

*a* adjusted p-values were calculated using Bonferroni’s and Benjamini–Hochberg corrections.

In a comparison between high-prevalence L4.2.2.2 and low-prevalence groups, 13 genes were identified with associations to either the highly prevalent L4.2.2.2 sublineage or the less prevalent group. Ten of these genes were non-annotated hypothetical protein-coding genes, and, except for the *Rv0025* gene, all other 12 genes showed significant associations with the low-prevalence phenotype (Table 2).

**Table 2:** MTB L4’s genes associated with L4.2.2.2 or low prevalent sublineages identified in GWAS analysis.

Gene	Uniprot code	Length (aa)	Annotation	Genomes number high prev. N = 20	Genomes number low prev. N = 29	P-value	Bonferroni’s-adjusted P-value^a^	Benjamini-H. adjusted P-value^a^	Genes association
***Rv2717c***	P9WFG7	164	Hypothetical protein	0	27	8.17E-12	1.35E-08	1.68E-09	LP
***Rv0021c***	P71591	322	Hypothetical protein	4	29	1.45E-09	2.38E-06	1.99E-07	LP
***narX/ Rv1736c***	P9WJQ1	652	Nitrate reductase-like protein	5	29	9.84E-09	1.62E-05	9.004E-07	LP
***arsB1***	P9WPD7	428	Arsenic-transport integral membrane protein	0	23	1.63E-08	2.69E-05	1.42E-06	LP
***Rv0654***	P9WPR5	501	Carotenoid cleavage oxygenase	0	22	3.29E-08	5.42E-05	1.59E-06	LP
***Rv0075***	O53620	390	Aminotransferase	0	22	3.29E-08	5.42E-05	1.59E-06	LP
***Rv0073***	P9WQK5	330	Glutamine ABC transporter ATP-binding protein	0	22	3.29E-08	5.42E-05	1.59E-06	LP
***Rv1132***	O06583	576	Conserved membrane protein	0	22	3.29E-08	5.42E-05	1.59E-06	LP
***eccC4***	P9WNA7	1236	ESX-4 secretion system protein	0	22	3.29E-08	5.42E-05	1.59E-06	LP
***Rv3047c***	I6X642	94	Hypothetical protein	0	22	3.29E-08	5.42E-05	1.59E-06	LP
***TB27*.*3***	P9WIR3	261	Conserved protein TB27.3	0	22	3.29E-08	5.42E-05	1.59E-06	LP
***PE_PGRS42***	I6XEF1	694	PE-PGRS family protein	0	21	5.04E-07	0.0008	1.85E-05	LP
***Rv0025***	P9WMA1	120	Hypothetical protein	20	15	0.0002	0.32	0.004	HP

*HP* high prevalence; *LP* low prevalence

*a* adjusted p-values were calculated using Bonferroni’s and Benjamini–Hochberg corrections.

### Mutations associated with the prevalence

In the comparative genomic analysis between L4.6.3 and the less prevalent group, ten genetic variants (indel and SNPs) were identified, reaching statistical significance with a p-value < 0.05 after Benjamini-H correction. These variants resulted in the inactivation, truncation, or deletion of genes where they were located. Specifically, these variants were distributed across four coding DNA sequences (CDS) of the highly prevalent L4.6.3 and seven CDS of the low-prevalence group, as detailed in S3 Table. Furthermore, the study observed an inverse association between the presence of genetic variants (indels, and SNPs) and the presence of genes. The six variants that exhibited a significant association with highly prevalent L4.2.2.2 traits were identified as deletions. These deletions occurred in the following genes: *Rv2717c* at position g.148-165del, *arsB1* at position g.1104delC, *Rv0075* at position g.836delC, *Rv0073* at position g.763delT, *Rv1132* at position g.502delG, and *Rv3047c* at position g.3delC, as outlined in S4 Table.

**Fig 6:**
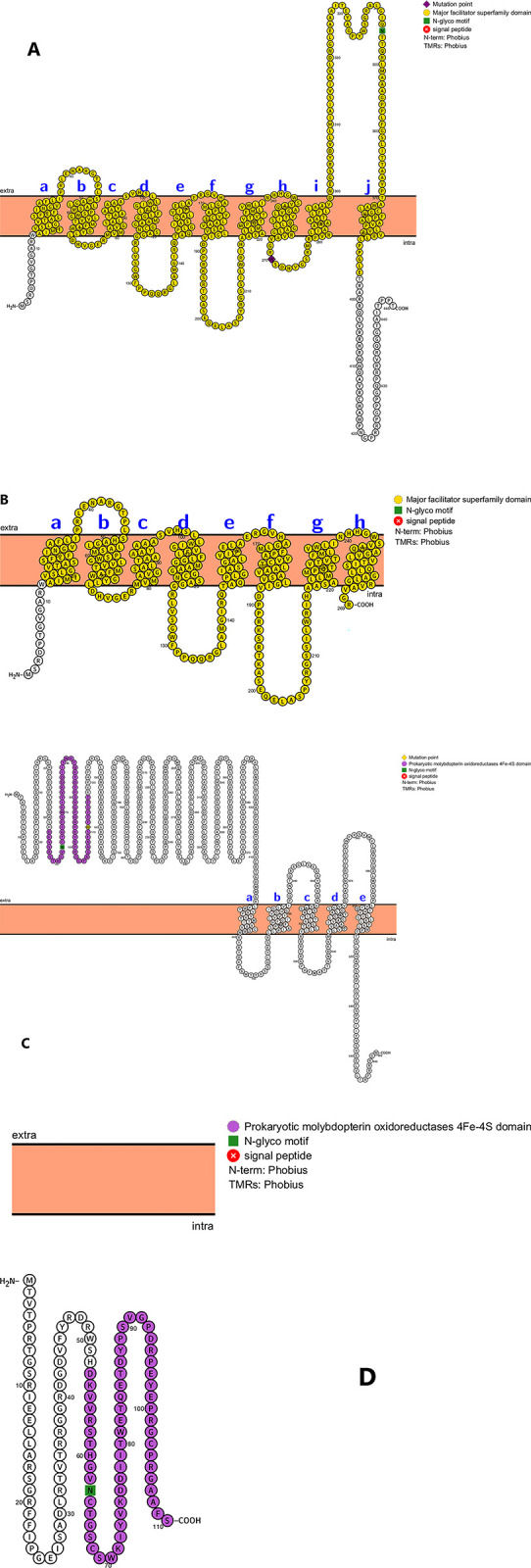
Secondary structure and cellular localization of the predicted membrane proteins *Rv2994* and *NarX*, utilizing Protter server v1.0 [31]. (**A**) The complete protein of *Rv2994* in low prevalence isolates with 445 amino acids and (**B**) its truncated protein in high prevalence L4.6.3 with 269 amino acids, codon stop at position 270. (**C**) Similarly, the *narX* gene is complete in low prevalence isolates with 652 amino acids and (**D**) but is truncated in the high prevalence L4.2.2.2 group with 110 amino acids, codon stops at position 111.

The significance of these variants associated with either high or low-prevalence isolates was confirmed through various bioinformatics tools. For example, the study visualized the gene locus of *Rv2994* and *narX*, where a single coding DNA sequence (CDS) associated with low-prevalence isolates was observed to be truncated into two CDS due to SNP mutations in high-prevalence L4.6.3 and L4.2.2.2 genomes, respectively (Fig 6). This type of mutation, along with others, generally leads to the inactivation of the genes in which they are located (S6–S9 Figs).

## Discussion

Genomic-wide association studies have sought to identify genetic determinants of MTB strains that could elucidate why certain L4 MTB strains exhibit higher dominance over other strains. However, such genetic determinants have not been identified for MTB strains causing EPTB in Ethiopia. To the best of our knowledge, this is the first pan-genomic and GWAS analysis conducted on L4 MTB in Ethiopia.

### Pan-genome of L4 of MTB strains

Constructing a pan-genome is crucial as it greatly facilitates gene discovery and contributes to our understanding of the genome architecture within a species. This, in turn, helps us identify the best vaccine-candidate antigens, drug target genes, and genes involved in bacterial virulence. A pan-genome encompasses the entire genomic repertoire of a species, allowing for the exploration of questions related to the diverse phenotypes observed by individuals of that species [12]. The current pan-genome analysis has facilitated the definition of a conserved core genome and a dynamic accessory genome of MTB isolates. The entire set of 5277 gene clusters (pangenome) from the 75 genomes was categorized into the core genome, comprising 3270 genes, and the accessory genome, comprising 2007 genes. Previous pan-genome studies identified a similar, albeit slightly higher, number of core genes when utilizing a significantly smaller number of MTB genomes [38, 39]. As anticipated, with the addition of each new genome to the analysis, the size of the core genome exhibited a decrease.

The results of this study also indicate that the pan-genome of L4 MTB strains is open, consistent with previous studies [38–41]. Moreover, through the analysis of a significant number of genomes using robust bioinformatics tools, this study supports the conclusion that MTB genomes possess an open nature, enabling the integration of new genes into their global repertoire. The mechanisms of gene duplication and fission likely play roles in the acquisition of new genes by MTB [42]. The pan-genome’s size is also influenced by gene loss; if the pathogen’s niche changes, some genes may be used less and eventually lost [38]. This biological flexibility, indicated by an open genome, suggests that the accessory genome of MTB could play a crucial role in adaptive responses and intra-species diversity at the sublineage level.

### Functional analysis of the MTB L4 pangenome

The data from this study support the notion that core genes constitute the largest fraction of the pan-genome. These core genes are considered primary genes and encompass the majority of genes essential for the survival, growth, and reproduction of MTB [43, 44]. For instance, in this study about 8% of core genes identified were associated with functions in the ‘[I] Lipid transport and metabolism’ category, which ranked second amongst the COG categories after the ‘[R] General function prediction only’ category. Lipid enrichment is a fundamental and crucial biological characteristic of MTB, given that a significant portion of lipids are located in the MTB cell wall, constituting around 40% of the cellular dry mass [45, 46]. This highlighting of lipid-related functions aligns with the importance of lipids in the biology of L4 MTB. They have also been demonstrated to be involved in functions related to high virulence, evasion of host immune responses, cell invasion, and slow growth [45, 46]. This study’s findings align with similar observations in other mycobacterial strains, such as *Mycobacterium abscessus*, where core genes were found to be crucial for survival, indicating the generality of these observations across different *Mycobacterial* species [47].

Our study results also revealed a total of 1667 genes identified in the accessory genome, comprising dispensable genes located in the shell compartment and unique genes found in the cloud. These dispensable genes and strain-specific genes are categorized as secondary genes, delineating the partially shared and strain-specific attributes of a species. These characteristics distinguish strains from one another and contribute to species diversity [43, 48]. Partially shared and strain-specific genes play roles that are not essential for growth but provide selective advantages, such as adaptation to different hosts and antibiotic resistance [43]. For instance, studies have shown that the L2 is characterized by a higher transmission frequency compared to other lineages, possibly due to shell genes, in addition to being associated with a higher drug resistance, which suggests relatively high transmission fitness [49, 50].

Similarly, in this study, COG analysis revealed that a significant proportion of unique genes were associated with functions related to ‘Cell motility (COG5651, PPE protein)’ and ‘Secondary metabolites biosynthesis, transport, and catabolism’. The COG5651/PPE repeat protein family, identified in the ‘Cell motility’ category, is known to be a major source of antigenic variability among different isolates [51]. Proteins belonging to this family play a key role in modulating host immune responses, resisting various stresses imposed by the host (such as low pH, hypoxia, reactive oxygen species, nutrient starvation, and antimicrobial drugs), and manipulating host cell fates (inhibiting apoptosis and autophagy and inducing necrosis) [52]. Overall, these functions contribute to the adaptability of MTB, highlighting the importance of dispensable and strain-specific genes in species diversity and environmental adaptation.

### GWAS of MTB L4

#### Genes associated with the highly prevalent L4.6.3 and L4.2.2.2 isolates

Point mutations in genes associated with drug resistance are well-established contributors to drug resistance in MTB. However, other mechanisms, such as drug efflux pumps and changes in cell wall permeability, also play crucial roles in drug tolerance [53]. For example, certain MTB strains, such as the Beijing/W strains, exhibit the RD105 deletion affecting the *Rv0068-Rv0075* genes, resulting in a fused *Rv0068/75* gene [53–56]. This fusion has been linked to increased resistance to multiple drugs by thickening the cell wall, thereby reducing the intracellular concentration of antibiotics [53–56]. In the current study, deletions were observed in the *Rv0071* gene in the L4.6.3 sublineage (at position g.514-518delCGGCT) and in the *Rv0075* and *Rv0073* genes in the L4.2.2.2 sublineage (at positions g.836delC and g.763delT, respectively). These deletions could potentially contribute to pathogenicity and drug resistance characteristics in these sublineages. This suggests that, besides point mutations, structural variations like deletions in specific genes might play a role in the development of pathogenic and drug-resistant phenotypes in different MTB sublineages.

The Type VII secretion system (T7SS) in MTB consists of five subtypes, known as ESX-1 to ESX-5 [57]. In this study, we identified SNPs at positions p.Glu279_ (gag/Tag) and p.His89Arg (cac/cGc) in the *eccC4* gene, which encodes a membrane protein essential for ESX-4, within L4.2.2.2 genomes. These SNPs cause a frameshift in the open reading frame, leading to the truncation of the *eccC4* gene. This likely renders the gene non-functional and it may eventually be deleted from the L4 genomes. The deletion of *eccC4*, a critical component of ESX-4, has been associated with increased secretion of protein substrates of ESX-1 and ESX-5 [57]. ESX-1 is known to mediate phagosome rupture inside macrophages, enabling the bacteria to escape from the phagolysosome, and it plays a crucial role in MTB virulence [58]. On the other hand, ESX-5 is essential for iron and fatty acid uptake [59] and is involved in modulating the host’s immune response [60]. These findings suggest that genetic variations in the T7SS, such as the observed SNP and potential loss of function in the *eccC4* gene, could influence the secretion of proteins associated with virulence and immune modulation in MTB L4.2.2.2 sublineages.

It has been previously demonstrated that induction of autophagy can suppress the intracellular survival of mycobacteria, and the *PE_PGRS20* family protein of MTB has been proposed to act as an inhibitor of autophagy to promote mycobacterial survival [61]. The *PE_PGRS20* family protein of MTB has been suggested to act as an autophagy inhibitor, promoting mycobacterial survival [61]. In our study, the *PE-PGRS20* gene, a substrate of ESX-5, was found to be significantly associated with L4.6.3 high-prevalence genomes. However, it exhibited a SNP in low-prevalence isolates at position p.Thr218Ser (acg/Tcg), resulting in a non-synonymous mutation that truncates the protein at position 120. These mutants showed increased autophagy and reduced intracellular survival in macrophages [61], potentially contributing to their lower prevalence compared to L4.6.3 strains. The study also highlighted the role of *Rv1928c*, one of the L4.6.3-associated genes, in aiding MTB survival and reproduction in macrophage phagosomes under conditions of low pH, oxygen, and starvation [62]. Although the precise roles of *Rv3093c* and *Rv0025* genes in MTB pathogenesis are not clearly defined, transcriptional analysis has indicated their involvement in the pathogenic processes of MTB [57, 63].

#### Genes associated with low prevalent isolates

MTB enters a non-replicating state, or dormancy when exposed to various stress conditions such as low oxygen tension, low pH, nutrient starvation, oxidative stress, DNA damage, transcription inhibition, iron scavengers, minimal medium (with succinate as a carbon source), and other factors encountered in granulomas during infection [64]. In our study, although the functions of some genes remain unknown, previous research has revealed their involvement in persistence (or dormancy). These genes include *Rv2717c* (involved in DNA repair and cell division arrest [64], *icl2* (linked to nutrient starvation) [65], *fadD34* (associated with nutrient starvation) [66], *Rv0021c* (related to nutrient starvation and oxygen-limiting conditions) [67], *NarX* (responsive to low oxygen tension) [68], *Rv0654* (affected by high temperature) [69], *Rv1132* (influenced by low pH) [70], and *Rv3047c* (responsive to low oxygen tension) [71], were significantly associated with low-prevalent isolates but exhibited different indels and SNPs in highly prevalent L4.6.3 and L4.2.2.2 isolates. This suggests that high-prevalent isolates exhibit a reduced ability to enter a state of latency, leading to a higher incidence of active TB cases and contributing to increased transmission of these isolates.

Moreover, the functions of *PE_PGRS6*/*Rv0532* [72], *Rv2994* [73], *arsB1*/*Rv2685* [74], *TB27*.*3*/*Rv0577* [75], and *PE_PGRS42* [76] are not fully understood, but previous studies have identified them as promising antigens/epitopes for a TB vaccine. In this study, these genes exhibited different polymorphisms and were associated with L4.6.3 or L4.2.2.2, the highly prevalent sublineages, unlike in low-prevalence isolates. These genetic variants may represent a mechanism for evading recognition by the host immune system, providing a selective advantage for highly prevalent isolates in the region. For example, *Rv2994*, a predicted transmembrane protein involved in the efflux system with known epitopes, gained a stop codon [77]. Additionally, it has been suggested that the PE/PPE gene family encodes virulence factors and is a possible source of antigenic variation influencing immune evasion [78].

In this study, a novel toxin-antitoxin (TA) system termed mt-PemIK was identified in all low-prevalent strains of L4 MTB, consisting of the antitoxin mt-PemI and the toxin mt-PemK (*Rv3098A*). Similarly, the second TA system identified by our GWAS in all low-prevalent strains was the VapBC20 TA system, where *VapB20* functions as the antitoxin, and *VapC20* acts as the toxin. Under normal conditions, VapB and VapC interact to form a hetero-octameric complex that inhibits their expression [79]. However, under stress conditions, the VapB antitoxin is degraded by cellular proteases, releasing free VapC toxin, arresting the growth of MTB, and leading to persistence. We observed that the mutation of g.214-215insTC in *Rv3098A* and the fusion of misc_RNA to *vapC28* genes in the high-prevalence L4.6.3 strains abrogated the toxicity associated with the wild-type (low-prevalence) strain. Thus, high-prevalence L4.6.3 strains lacking the *Rv3098A* and *vapC28* genes may exhibit a distinct phenotype characterized by fast cell growth, enhancing their ability to escape from the lungs and spread more rapidly. Consistent with our study, overexpression of wild-type *Rv3098A* [80] and *vapC28* [81] toxins resulted in bacteriostasis, whereas no growth inhibition was observed in strains overexpressing mutant proteins.

## Conclusions and recommendations

The results of this study revealed significant genomic differences between high and low-prevalence strains of the sublineages of L4 of MTB. The housekeeping core genome, representing a substantial portion of the pan-genome, holds promise for further exploration in diagnostics, drug target identification, and vaccine development. The observation of an open pan-genome in L4 of MTB suggests the potential for the acquisition or loss of genes in response to environmental challenges, contributing to its adaptability. Furthermore, various virulence factors associated with the spread of L4.6.3 and L4.2.2.2 were identified. While the association of these genes and their genetic variants with MTB transmission has been confirmed using different bioinformatic tools, experimental validation is needed. Hence, it is recommended to conduct animal model trials to investigate the roles of these genes in the pathogenicity and transmission of specific L4 MTB sublineages over other L4 MTB sublineages.

## Supporting information

S1 Table Patient characteristics, isolates prevalence category and sequencing read features (N = 75). (DOCX)

S2 Table Scaffold’s features, annotated genes, and quality of the genome assembly (N = 75). (DOCX)

S3 Table Mutations associated with L4.6.3 (N = 26) or low prevalence (N = 29) of MTB lineage-4 in western Ethiopia. (DOCX)

S4 Table Mutations associated with L4.2.2.2 (N = 20) or low prevalence (N = 29) of MTB lineage-4 in western Ethiopia. (DOCX)

S1 Fig Prevalence of MTB sublineage-4 in western Ethiopia. A histogram denoting the distribution of MTB L4 sublineages and the numbers on top of the bars indicate the percentage of sub-lineages. Except for L4.6.3 and L4.2.2.2, all other sub-lineages of L4 were classified as low prevalent groups. *L* lineage.
(TIF)

S2 Fig Global annotation of the 75 MTB genome. Average of CDSs annotated by gene prediction and homology of sequence, 2,880 CDSs (72.24) had a functional assignment in the annotation, 1,122(26.48%) corresponded to hypothetical proteins, and 52 (1.28) to tRNA.
(TIF)

S3 Fig Whole genome multilocus sequence typing (wgMLST) based phylogeny. The outgroup was *M*. *canetti* CIPT 140010059 and the shapes on each tip of all branches indicate sub-lineages of L4. The red shades correspond to isolates with a high prevalence of L4.2.2.2 sub-lineage, the light purple corresponds high prevalence of L4.6.3, and the yellow shades correspond to all isolates with a low prevalence in western Ethiopia.
(TIF)

S4 Fig Pangeneome of MTB Lineage-4. The intersection of COG and OMCL algorithms is the total number of gene clusters of which it is composed of the set 75 genomes.
(TIF)

S5 Fig Core Genome of MTB Lineage 4. The intersection of three algorithms of the cluster of orthologous genes. In the center, the number of clusters shared in 100% of the isolates. Unique clusters were identified by BDBH (1), OMLC (4), and COG (60) algorithms. Some gene clusters are observed shared between two of the three algorithms (script *comare_clusters*.*pl* from get_homologues).
(TIF)

S6 Fig Comparative analysis with parSNP. The left side shows the core phylogeny of 75 isolates using H37Rv as a reference (GenBank accession number NC_000962.3). The right side corresponds to a multi-genomic alignment against the phylogenetic tree. The red letter (A) in the white box shows the variant (SNP) in the *fadD34* gene of high prevalence L4.6.3 genomes.
(TIF)

S7 Fig Comparative analysis with Parsnp. The left side shows the core phylogeny of 75 isolates using H37Rv as a reference (GenBank accession number NC_000962.3). The right side corresponds to a multi-genome alignment against the phylogenetic tree. The red letter (A) in the white box shows the variant (SNP) in the *Rv0021c* gene of high prevalence L4.2.2.2 genomes.
(TIF)

S8 Fig Comparative analysis with Mauve. The joined segmented rectangles or squares correspond to the CDS annotated. The blue-shaded rectangle shows the locus annotation for the *Rv1928c* (**A**) and *arsB1* (**B**) genes in H37Rv, reference genome (GenBank accession number NC_000962.3). (**A**) The red arrow in the red box shows the variant difference of the *Rv1928c* gene between high prevalence L4.6.3 genomes with a complete CDS and low prevalence with two smaller CDS are observed, the first with a premature stop codon due to deletion and the second after the deletion despite having a start codon possibly not be functional. (**B**) The red arrow in the red box shows the genetic variant difference of the *arsB1* gene between low prevalence genomes with a complete CDS and high prevalence L4.2.2.2 with two smaller CDS are observed, the first with a premature stop codon due to deletion and the second after the deletion despite having a start codon possibly not be functional.
(TIF)

S9 Fig Comparative analysis by blastn against H37Rv as a reference (GenBank accession number NC_000962.3). The gap in the red circle shows the insertion in the *Rv3098A* (**A**) and deletion in the *Rv0071c* (**B**) genes of high prevalence L4.6.3 genomes.
(TIF)

S1 File (DOCX)
