# Supplementary material for: Pangenome and genomic signatures linked to the dominance of the lineage-4 of Mycobacterium tuberculosis isolated from extrapulmonary tuberculosis patients in western Ethiopia
Source: PLoS One. 2024 Jul 25;19(7):e0304060. doi: 10.1371/journal.pone.0304060 (PMC11271921; doi:10.1371/journal.pone.0304060)
Supplement: S1 Table — (DOCX) [file pone.0304060.s001.docx]

**S1 Table. Patient characteristics, isolates prevalence category and sequencing read features (N=75)**

| No. | ID | Age | Sex | Prevalence | Total reads | % Mapped reads | Coverage mean | Total bases | %Total bases |
| --- | --- | --- | --- | --- | --- | --- | --- | --- | --- |
| 1 | EN001 | 65 | F | Low | 8116305 | 99.48 | 237.61 | 4398015 | 1 |
| 2 | EN012 | 18 | M | L4.2.2.2 | 4349942 | 96.41 | 121.29 | 4390415 | 1 |
| 3 | EN013 | 65 | M | L4.2.2.2 | 7385239 | 98.69 | 213.73 | 4391993 | 1 |
| 4 | EN015 | 12 | M | Low | 35649021 | 98.9 | 1032.33 | 4401365 | 1 |
| 5 | EN019 | 32 | F | L4.6.3 | 25828349 | 99.69 | 763.76 | 4405040 | 1 |
| 6 | EN020 | 45 | M | L4.2.2.2 | 6802589 | 69.97 | 142.72 | 4388414 | 0.99 |
| 7 | EN021 | 12 | M | L4.2.2.2 | 8506475 | 99.42 | 252.83 | 4391965 | 1 |
| 8 | EN022 | 30 | F | Low | 7038627 | 99.74 | 209.77 | 4367695 | 0.99 |
| 9 | EN023 | 9 | M | Low | 6724012 | 99.5 | 197.22 | 4397282 | 1 |
| 10 | EN027 | 18 | F | L4.6.3 | 8004594 | 91.92 | 212.68 | 4401172 | 1 |
| 11 | EN030 | 21 | F | L4.6.3 | 5606616 | 98.23 | 159.6 | 4402357 | 1 |
| 12 | EN034 | 15 | F | Low | 18676028 | 97.67 | 540.75 | 4400642 | 1 |
| 13 | EN035 | 45 | F | L4.6.3 | 7538891 | 99.09 | 218.22 | 4401063 | 1 |
| 14 | EN036 | 60 | F | Low | 10172739 | 99.14 | 291.5 | 4396314 | 1 |
| 15 | EN037 | 27 | F | Low | 8389876 | 98.73 | 247.01 | 4393361 | 1 |
| 16 | EN038 | 27 | F | L4.6.3 | 8281340 | 99.05 | 232.6 | 4403080 | 1 |
| 17 | EN041 | 25 | M | Low | 7047354 | 99.62 | 201.62 | 4408366 | 1 |
| 18 | EN042 | 18 | F | L4.6.3 | 9231478 | 99.58 | 263.59 | 4404124 | 1 |
| 19 | EN043 | 30 | M | L4.2.2.2 | 6603590 | 99.33 | 193.17 | 4387973 | 0.99 |
| 20 | EN045 | 48 | M | Low | 14169589 | 83.82 | 339.97 | 4398971 | 1 |
| 21 | EN046 | 25 | F | Low | 6532229 | 62.49 | 116.58 | 4396037 | 1 |
| 22 | EN048 | 15 | F | Low | 4152284 | 98.13 | 117.67 | 4389736 | 1 |
| 23 | EN052 | 15 | F | L4.2.2.2 | 6789102 | 99.28 | 199.75 | 4390154 | 1 |
| 24 | EN054 | 32 | M | L4.6.3 | 8744575 | 99.63 | 253.59 | 4400949 | 1 |
| 25 | EN058 | 21 | F | Low | 9443990 | 92.01 | 254.79 | 4390284 | 1 |
| 26 | EN059 | 22 | F | Low | 9220887 | 99.78 | 270.42 | 4357257 | 0.99 |
| 27 | EN062 | 47 | F | Low | 9083716 | 99.49 | 266.56 | 4394530 | 1 |
| 28 | EN064 | 35 | M | L4.2.2.2 | 8340493 | 99.46 | 242.99 | 4391527 | 1 |
| 29 | EN100 | NA | M | L4.6.3 | 2258206 | 99.73 | 63.38 | 4397033 | 1 |
| 30 | EN105 | 17 | M | L4.6.3 | 1322877 | 99.45 | 36.32 | 4399584 | 1 |
| 31 | EN108 | NA | F | Low | 1607458 | 99.2 | 44.1 | 4390279 | 1 |
| 32 | EN144 | 33 | M | Low | 1162067 | 84.5 | 26.79 | 4389104 | 0.99 |
| 33 | EN145 | 51 | F | L4.6.3 | 1285045 | 99.71 | 35.54 | 4393011 | 1 |
| 34 | EN146 | 26 | F | L4.2.2.2 | 1566468 | 99.28 | 42.77 | 4383835 | 0.99 |
| 35 | EN148 | 20 | F | L4.6.3 | 9438563 | 68.58 | 180.72 | 4405531 | 1 |
| 36 | EN150 | 31 | M | L4.6.3 | 20106500 | 99.37 | 563.42 | 4404392 | 1 |
| 37 | EN154 | 45 | M | L4.2.2.2 | 9100210 | 99.44 | 250.19 | 4393151 | 1 |
| 38 | EN155 | 29 | M | L4.6.3 | 1013758 | 99.56 | 28.43 | 4383657 | 0.99 |
| 39 | EN158 | 18 | F | L4.6.3 | 2329835 | 99.6 | 66.15 | 4396839 | 1 |
| 40 | EN161 | 28 | M | L4.2.2.2 | 4233128 | 99.36 | 116.49 | 4390648 | 1 |
| 41 | EN162 | 52 | M | L4.2.2.2 | 2903662 | 99.08 | 78.94 | 4388039 | 0.99 |
| 42 | EN244 | 43 | F | L4.6.3 | 7046065 | 97.43 | 191.89 | 4405743 | 1 |
| 43 | EN248 | 18 | F | L4.6.3 | 3254972 | 98.31 | 88.99 | 4400025 | 1 |
| 44 | EN251 | 17 | F | Low | 1309692 | 99.47 | 35.86 | 4389422 | 0.99 |
| 45 | EN260 | 27 | F | Low | 1530074 | 99.46 | 42.72 | 4394234 | 1 |
| 46 | EW008 | 18 | F | L4.6.3 | 6282292 | 77.64 | 142.56 | 4398394 | 1 |
| 47 | EW009 | 20 | M | Low | 7871616 | 98.78 | 225.64 | 4399417 | 1 |
| 48 | EW010 | 21 | F | Low | 5937751 | 78.33 | 135.83 | 4391710 | 1 |
| 49 | EW011 | 26 | F | Low | 6436282 | 99.19 | 185.45 | 4394208 | 1 |
| 50 | EW069 | 25 | M | Low | 2784627 | 99.53 | 77.37 | 4396096 | 1 |
| 51 | EW070 | 2 | M | L4.2.2.2 | 3120925 | 99.1 | 88.76 | 4386423 | 0.99 |
| 52 | EW071 | 30 | F | L4.2.2.2 | 781787 | 99.4 | 21.77 | 4379774 | 0.99 |
| 53 | EW072 | 26 | M | L4.6.3 | 1143230 | 99.74 | 32.63 | 4393711 | 1 |
| 54 | EW073 | 35 | M | Low | 1477413 | 99.56 | 40.92 | 4386707 | 0.99 |
| 55 | EW074 | 50 | M | L4.2.2.2 | 900016 | 99.42 | 25.62 | 4385769 | 0.99 |
| 56 | EW075 | 24 | F | Low | 1995478 | 99.4 | 56.51 | 4391816 | 1 |
| 57 | EW078 | 48 | F | L4.2.2.2 | 1821411 | 99.42 | 50.91 | 4385499 | 0.99 |
| 58 | EW079 | 45 | M | L4.6.3 | 862587 | 99.74 | 23.93 | 4395951 | 1 |
| 59 | EW083 | 65 | M | Low | 3727657 | 99.52 | 103.42 | 4403330 | 1 |
| 60 | EW087 | 15 | F | L4.6.3 | 4424555 | 99.73 | 124.47 | 4398777 | 1 |
| 61 | EW090 | 19 | F | L4.6.3 | 1500162 | 99.33 | 41.75 | 4395009 | 1 |
| 62 | EW094 | 26 | F | Low | 4084790 | 99.43 | 116.67 | 4398011 | 1 |
| 63 | EW110 | NA | NA | L4.2.2.2 | 833098 | 99.25 | 23.36 | 4375381 | 0.99 |
| 64 | EW114 | 18 | F | Low | 1051504 | 99.52 | 28.81 | 4382909 | 0.99 |
| 65 | EW117 | NA | NA | L4.6.3 | 1721966 | 99.59 | 48.24 | 4386702 | 0.99 |
| 66 | EW118 | 35 | F | L4.2.2.2 | 1939649 | 99.3 | 53.95 | 4388022 | 0.99 |
| 67 | EW122 | 23 | M | L4.6.3 | 1754680 | 91.25 | 43.83 | 4400200 | 1 |
| 68 | EW124 | 46 | M | L4.6.3 | 849313 | 99.51 | 23.73 | 4393687 | 1 |
| 69 | EW125 | 27 | M | L4.6.3 | 2203826 | 99.64 | 61.89 | 4401167 | 1 |
| 70 | EW126 | NA | NA | L4.2.2.2 | 1527611 | 99.44 | 41.13 | 4386455 | 0.99 |
| 71 | EW127 | 18 | M | L4.2.2.2 | 1416113 | 98.82 | 38.78 | 4396618 | 1 |
| 72 | EW133 | 50 | M | Low | 1989244 | 98.6 | 56.67 | 4393598 | 1 |
| 73 | EW138 | 13 | M | Low | 1832848 | 99.28 | 49.58 | 4403677 | 1 |
| 74 | EW185 | 25 | M | L4.2.2.2 | 3361815 | 99.45 | 91.54 | 4389275 | 0.99 |
| 75 | EW199 | 30 | F | L4.6.3 | 2207063 | 96.71 | 60.07 | 4402657 | 1 |
| Average | |  |  |  | **5676530.92** | **96.632** | **157.3708** | **4393674.11** | **0.9973** |

*NA* not available, *Low* isolates categorized in the low prevalent group
