# Supplementary material for: Pangenome and genomic signatures linked to the dominance of the lineage-4 of Mycobacterium tuberculosis isolated from extrapulmonary tuberculosis patients in western Ethiopia
Source: PLoS One. 2024 Jul 25;19(7):e0304060. doi: 10.1371/journal.pone.0304060 (PMC11271921; doi:10.1371/journal.pone.0304060)
Supplement: S2 Table — (DOCX) [file pone.0304060.s002.docx]

**S2 Table. Scaffold’s features, annotated genes, and quality of the genome assembly (N=75)**

| No. | ID | N50(bp) | Largest contig | No. contigs | Total length | Genome fraction (%) | G+C_% | CDS | tRNA | Compl  eteness | Contam  ination | Strain  Hetero  geneity |
| --- | --- | --- | --- | --- | --- | --- | --- | --- | --- | --- | --- | --- |
| 1 | EN001 | 83307 | 228341 | 112 | 4356767 | 97.74 | 65.5 | 4054 | 53 | 100 | 0 | 0 |
| 2 | EN012 | 65893 | 230068 | 125 | 4354664 | 97.65 | 65.4 | 4085 | 52 | 100 | 0 | 0 |
| 3 | EN013 | 64599 | 229989 | 108 | 4368835 | 97.9 | 65.4 | 4058 | 57 | 100 | 0 | 0 |
| 4 | EN015 | 80705 | 213145 | 101 | 4404978 | 97.92 | 65.4 | 4093 | 55 | 100 | 0 | 0 |
| 5 | EN019 | 84352 | 222753 | 102 | 4356067 | 98.16 | 65.4 | 4078 | 53 | 100 | 0 | 0 |
| 6 | EN020 | 7232 | 230051 | 124 | 4353495 | 97.73 | 65.4 | 4059 | 53 | 100 | 0 | 0 |
| 7 | EN021 | 82712 | 230030 | 104 | 4352661 | 97.77 | 65.5 | 4057 | 53 | 100 | 0 | 0 |
| 8 | EN022 | 79191 | 202329 | 114 | 4319615 | 97.32 | 65.4 | 4040 | 53 | 100 | 0 | 0 |
| 9 | EN023 | 82284 | 228390 | 116 | 4358141 | 97.77 | 65.5 | 4066 | 53 | 100 | 0 | 0 |
| 10 | EN027 | 19688 | 222619 | 122 | 4359640 | 97.91 | 65.5 | 4073 | 53 | 100 | 0 | 0 |
| 11 | EN030 | 71018 | 222452 | 123 | 4360087 | 97.97 | 65.4 | 4069 | 53 | 100 | 0 | 0 |
| 12 | EN034 | 64021 | 226585 | 105 | 4340499 | 97.73 | 65.4 | 4072 | 53 | 100 | 0 | 0 |
| 13 | EN035 | 84001 | 222660 | 112 | 4351428 | 97.94 | 65.5 | 4069 | 53 | 100 | 0 | 0 |
| 14 | EN036 | 59426 | 228073 | 139 | 4372516 | 97.85 | 65.4 | 4096 | 53 | 100 | 0 | 0 |
| 15 | EN037 | 84089 | 223401 | 105 | 4360389 | 97.91 | 65.5 | 4070 | 53 | 100 | 0 | 0 |
| 16 | EN038 | 80914 | 197658 | 118 | 4350800 | 97.99 | 65.5 | 4058 | 53 | 100 | 0 | 0 |
| 17 | EN041 | 69543 | 222814 | 130 | 4389165 | 97.97 | 65.4 | 4137 | 53 | 100 | 0 | 0 |
| 18 | EN042 | 81489 | 192413 | 124 | 4350570 | 97.83 | 65.4 | 4090 | 53 | 100 | 0 | 0 |
| 19 | EN043 | 84476 | 230244 | 113 | 4358807 | 97.83 | 65.4 | 4083 | 53 | 100 | 0 | 0 |
| 20 | EN045 | 52390 | 205112 | 111 | 4369379 | 97.84 | 65.4 | 4086 | 52 | 100 | 0 | 0 |
| 21 | EN046 | 22537 | 226175 | 147 | 4373395 | 97.77 | 65.4 | 4102 | 53 | 100 | 0 | 0 |
| 22 | EN048 | 82262 | 222774 | 130 | 4332631 | 97.62 | 65.5 | 4059 | 53 | 100 | 0 | 0 |
| 23 | EN052 | 79227 | 229977 | 114 | 4355143 | 97.74 | 65.4 | 4064 | 52 | 100 | 0 | 0 |
| 24 | EN054 | 71197 | 222699 | 122 | 4351620 | 97.72 | 65.4 | 4096 | 53 | 100 | 0 | 0 |
| 25 | EN058 | 26700 | 159455 | 128 | 4354955 | 97.63 | 65.5 | 4063 | 53 | 100 | 0 | 0 |
| 26 | EN059 | 97788 | 223066 | 109 | 4302144 | 96.8 | 65.4 | 4036 | 52 | 100 | 0 | 0 |
| 27 | EN062 | 72379 | 223495 | 117 | 4353111 | 97.79 | 65.4 | 4076 | 53 | 100 | 0 | 0 |
| 28 | EN064 | 83858 | 230253 | 110 | 4358996 | 97.74 | 65.5 | 4061 | 53 | 100 | 0 | 0 |
| 29 | EN100 | 65782 | 222503 | 164 | 4335943 | 97.39 | 65.4 | 4100 | 52 | 100 | 0 | 0 |
| 30 | EN105 | 65752 | 188846 | 144 | 4332808 | 97.48 | 65.4 | 4052 | 52 | 100 | 0 | 0 |
| 31 | EN108 | 56290 | 178896 | 175 | 4333064 | 97.1 | 65.4 | 4150 | 52 | 100 | 0 | 0 |
| 32 | EN144 | 1185 | 158462 | 326 | 4408724 | 96.91 | 65.5 | 4173 | 53 | 100 | 2 | 0 |
| 33 | EN145 | 64158 | 158665 | 158 | 4330700 | 97.35 | 65.4 | 4091 | 53 | 100 | 0 | 0 |
| 34 | EN146 | 60826 | 230194 | 158 | 4337763 | 97.18 | 65.4 | 4110 | 52 | 97.67 | 0 | 0 |
| 35 | EN148 | 38258 | 222837 | 137 | 4401984 | 97.98 | 65.5 | 4113 | 56 | 100 | 0 | 0 |
| 36 | EN150 | 80970 | 203497 | 97 | 4360371 | 98.13 | 65.5 | 4053 | 57 | 100 | 0 | 0 |
| 37 | EN154 | 123769 | 207003 | 89 | 4365277 | 97.98 | 65.5 | 4044 | 53 | 100 | 0 | 0 |
| 38 | EN155 | 60507 | 193975 | 171 | 4305674 | 96.82 | 65.4 | 4051 | 53 | 100 | 0 | 0 |
| 39 | EN158 | 67989 | 157173 | 143 | 4334818 | 97.61 | 65.4 | 4104 | 52 | 100 | 0 | 0 |
| 40 | EN161 | 83851 | 230181 | 104 | 4358711 | 97.74 | 65.4 | 4085 | 53 | 100 | 0 | 0 |
| 41 | EN162 | 66116 | 230046 | 133 | 4354043 | 97.52 | 65.4 | 4086 | 53 | 100 | 0 | 0 |
| 42 | EN244 | 32504 | 222737 | 100 | 4360225 | 98 | 65.4 | 4074 | 53 | 100 | 0 | 0 |
| 43 | EN248 | 51670 | 162471 | 119 | 4358597 | 97.86 | 65.5 | 4103 | 55 | 100 | 0 | 0 |
| 44 | EN251 | 64161 | 228335 | 150 | 4337058 | 97.2 | 65.5 | 4079 | 53 | 100 | 0 | 0 |
| 45 | EN260 | 17055 | 224795 | 151 | 4343548 | 97.51 | 65.4 | 4122 | 54 | 100 | 0 | 0 |
| 46 | EW008 | 36191 | 222802 | 142 | 4344117 | 97.67 | 65.5 | 4081 | 53 | 100 | 0 | 0 |
| 47 | EW009 | 71028 | 193618 | 121 | 4358922 | 97.78 | 65.5 | 4077 | 54 | 100 | 0 | 0 |
| 48 | EW010 | 31976 | 226279 | 139 | 4351044 | 97.51 | 65.5 | 4100 | 53 | 100 | 0 | 0 |
| 49 | EW011 | 67990 | 223490 | 107 | 4360564 | 97.79 | 65.4 | 4085 | 54 | 100 | 0 | 0 |
| 50 | EW069 | 81381 | 228321 | 127 | 4352518 | 97.62 | 65.5 | 4074 | 53 | 100 | 0 | 0 |
| 51 | EW070 | 64186 | 184065 | 137 | 4345021 | 97.42 | 65.4 | 4074 | 53 | 100 | 0 | 0 |
| 52 | EW071 | 55894 | 227955 | 178 | 4332499 | 96.87 | 65.3 | 4121 | 53 | 100 | 0 | 0 |
| 53 | EW072 | 51636 | 216436 | 174 | 4326399 | 97.15 | 65.4 | 4080 | 53 | 100 | 0 | 0 |
| 54 | EW073 | 63593 | 155526 | 158 | 4343573 | 97.3 | 65.4 | 4122 | 53 | 100 | 0 | 0 |
| 55 | EW074 | 47687 | 197688 | 188 | 4333427 | 97.06 | 65.4 | 4117 | 53 | 100 | 0 | 0 |
| 56 | EW075 | 64600 | 202907 | 146 | 4337738 | 97.27 | 65.4 | 4078 | 52 | 100 | 0 | 0 |
| 57 | EW078 | 61982 | 230038 | 141 | 4340503 | 97.37 | 65.4 | 4089 | 53 | 100 | 0 | 0 |
| 58 | EW079 | 57841 | 141048 | 182 | 4322876 | 97.22 | 65.4 | 4118 | 52 | 100 | 0 | 0 |
| 59 | EW083 | 79304 | 222909 | 127 | 4358922 | 97.94 | 65.5 | 4101 | 53 | 100 | 0 | 0 |
| 60 | EW087 | 67989 | 222697 | 134 | 4345619 | 97.77 | 65.5 | 4076 | 53 | 100 | 0 | 0 |
| 61 | EW090 | 64197 | 141040 | 159 | 4340665 | 97.46 | 65.4 | 4093 | 53 | 100 | 0 | 0 |
| 62 | EW094 | 79287 | 229941 | 115 | 4362760 | 97.85 | 65.4 | 4122 | 53 | 100 | 0 | 0 |
| 63 | EW110 | 49661 | 230016 | 179 | 4327389 | 97.01 | 65.4 | 4104 | 53 | 100 | 0 | 0 |
| 64 | EW114 | 55055 | 158768 | 169 | 4325169 | 96.95 | 65.4 | 4081 | 53 | 100 | 0 | 0 |
| 65 | EW117 | 63794 | 156671 | 157 | 4332898 | 97.42 | 65.4 | 4086 | 54 | 100 | 0 | 0 |
| 66 | EW118 | 70926 | 230197 | 134 | 4344886 | 97.35 | 65.4 | 4092 | 53 | 100 | 0 | 0 |
| 67 | EW122 | 43755 | 154303 | 157 | 4353498 | 97.58 | 65.4 | 4091 | 53 | 100 | 0 | 0 |
| 68 | EW124 | 47826 | 141045 | 194 | 4319618 | 97.09 | 65.4 | 4092 | 53 | 100 | 0 | 0 |
| 69 | EW125 | 71022 | 222655 | 136 | 4351919 | 97.72 | 65.4 | 4108 | 53 | 100 | 0 | 0 |
| 70 | EW126 | 58980 | 179539 | 152 | 4347403 | 97.43 | 65.4 | 4100 | 53 | 100 | 0 | 0 |
| 71 | EW127 | 59835 | 166616 | 138 | 4363369 | 97.7 | 65.4 | 4111 | 52 | 100 | 0 | 0 |
| 72 | EW133 | 64161 | 230131 | 153 | 4366664 | 97.58 | 65.4 | 4129 | 53 | 100 | 0 | 0 |
| 73 | EW138 | 64118 | 158447 | 153 | 4359054 | 97.73 | 65.4 | 4140 | 53 | 100 | 0 | 0 |
| 74 | EW185 | 78989 | 229991 | 126 | 4359363 | 97.68 | 65.4 | 4082 | 53 | 100 | 0 | 0 |
| 75 | EW199 | 47223 | 175936 | 138 | 4342724 | 97.66 | 65.5 | 4065 | 55 | 100 | 0 | 0 |
| Average | | **63203** | **206249** | **136.87** | **4350412** | **97.59** | **65.43** | **4087** | **53.12** | **99.97** | **0.027** | **0** |
