# Supplementary material for: Pangenome and genomic signatures linked to the dominance of the lineage-4 of Mycobacterium tuberculosis isolated from extrapulmonary tuberculosis patients in western Ethiopia
Source: PLoS One. 2024 Jul 25;19(7):e0304060. doi: 10.1371/journal.pone.0304060 (PMC11271921; doi:10.1371/journal.pone.0304060)
Supplement: S3 Table — (DOCX) [file pone.0304060.s003.docx]

**S3 Table. Mutations associated with** **L4.6.3 (N=26) or low prevalence (N=29) of MTB lineage-4 in western Ethiopia.**

| **Gene** | **#Position** | **Variants or gene deletion** | **Genomes number high prev. N = 26** | **Genomes number low prev. N = 29** | **Benjamini-H. adjusted P-value** | **Mutations association** |
| --- | --- | --- | --- | --- | --- | --- |
| ***Rv0071*** | 79999-80003 | g.514-518delCGGCT | 26 | 0 | 7.22E-14 | HP |
| ***vapC28/Rv0609*** | - | - | 26 | 0 | 7.22E-14 | HP |
| ***Rv3098A*** | 3467819 | g.214-215insTC | 26 | 0 | 7.22E-14 | HP |
| ***icl2*** | - | - | 26 | 8 | 3.58E-07 | HP |
| ***Rv1928c*** | 2180818 | g.400delT | 0 | 21 | 3.58E-07 | LP |
| ***PE_PGRS20*** | 1191497 | p.Thr218Ser (acg/Tcg) | 0 | 18 | 1.46E-05 | LP |
| ***Rv3093c*** | 3462145 | g.619-620insGGCGC | 0 | 16 | 0.0001 | LP |
| ***fadD34*** | 37887 | g.629-630insC | 13 | 0 | 0.0003 | HP |
| ***PE_PGRS6/Rv0532*** | 624078 | p.Ala429Gly(gcg/gGg) | 13 | 0 | 0.0003 | HP |
|  | 624089 | p.Asn433Asp (aac/Gac) |  |  |  |  |
| ***Rv0025*** | 29483 | g.239delA | 0 | 14 | 0.0007 | LP |
| ***Rv2994*** | 3352078 | p.Trp270_ (tgg/tgA) | 10 | 0 | 0.005 | HP |

*HP* high prevalence, *LP* low prevalence
