# Supplementary material for: Pangenome and genomic signatures linked to the dominance of the lineage-4 of Mycobacterium tuberculosis isolated from extrapulmonary tuberculosis patients in western Ethiopia
Source: PLoS One. 2024 Jul 25;19(7):e0304060. doi: 10.1371/journal.pone.0304060 (PMC11271921; doi:10.1371/journal.pone.0304060)
Supplement: S4 Table — (DOCX) [file pone.0304060.s004.docx]

**S4 Table. Mutations associated with L4.2.2.2 (N=20) or low prevalence (N=29) of MTB lineage-4 in western Ethiopia.**

| **Gene** | **#Position** | **Variants/gene deletion** | **Genomes number high prev. N = 20** | **Genomes number low prev. N = 29** | **Benjamini-H. adjusted P-value** | **Mutations association** |
| --- | --- | --- | --- | --- | --- | --- |
| ***Rv2717c*** | 3030197-3030213 | g.148-165del | 20 | 2 | 1.68E-09 | HP |
|  | 3030214 | p.Thr50Ser (acg/Tcg) |  |  |  |  |
| ***Rv0021c*** | 26666 | p.Trp72_ (tgg/tgA) | 16 | 0 | 1.99E-07 | HP |
| ***NarX/ Rv1736c*** | 1963855 | p.Trp111_ (tgg/tAg) | 15 | 0 | 9.004E-07 | HP |
| ***arsB1/Rv2685*** | 3003085 | g.1104delC | 20 | 6 | 1.42E-06 | HP |
| ***Rv0654*** | 751297 | g.1299_3000insC | 20 | 7 | 1.59E-06 | HP |
| ***Rv0075*** | 84831 | g.836delC | 20 | 7 | 1.59E-06 | HP |
| ***Rv0073*** | 82438 | g.763delT | 20 | 7 | 1.59E-06 | HP |
| ***Rv1132*** | 1257826 | g.502delG | 20 | 7 | 1.59E-06 | HP |
| ***eccC4*** | 3867404 | p.Glu279_ (gag/Tag) | 20 | 7 | 1.59E-06 | HP |
|  | 3867973 | p.His89Arg(cac/cGc) | 20 | 0 | 1.01E-11 |  |
| ***Rv3047c*** | 3408302 | g.3delC | 20 | 7 | 1.59E-06 | HP |
| ***TB27.3/ Rv0577*** | 671406 | g.242-243insTC | 20 | 7 | 1.59E-06 | HP |
| ***PE_PGRS42*** | 2796214 | g.1172-1173insC | 20 | 8 | 1.85E-05 | HP |
| ***Rv0025*** | 29483 | g.239delA | 0 | 14 | 0.004 | LP |

*HP* high prevalence; *LP* low prevalence
