# Supplementary figures and images for: Pangenome and genomic signatures linked to the dominance of the lineage-4 of Mycobacterium tuberculosis isolated from extrapulmonary tuberculosis patients in western Ethiopia
Source: PLoS One. 2024 Jul 25;19(7):e0304060. doi: 10.1371/journal.pone.0304060 (PMC11271921; doi:10.1371/journal.pone.0304060)

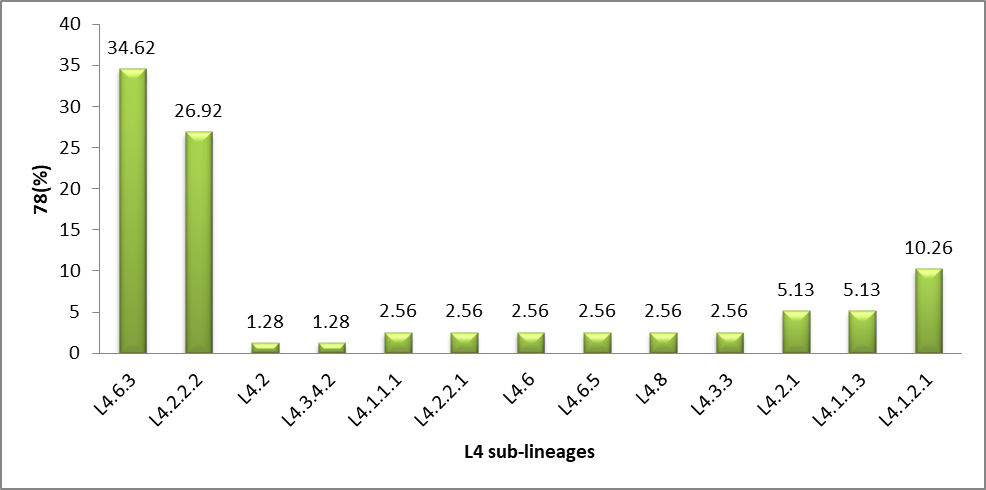

Supplement: S1 Fig — A histogram denoting the distribution of MTB L4 sublineages and the numbers on top of the bars indicate the percentage of sub-lineages. Except for L4.6.3 and L4.2.2.2, all other sub-lineages of L4 were classified as low prevalent groups. L lineage. (TIF) [file pone.0304060.s005.tif]

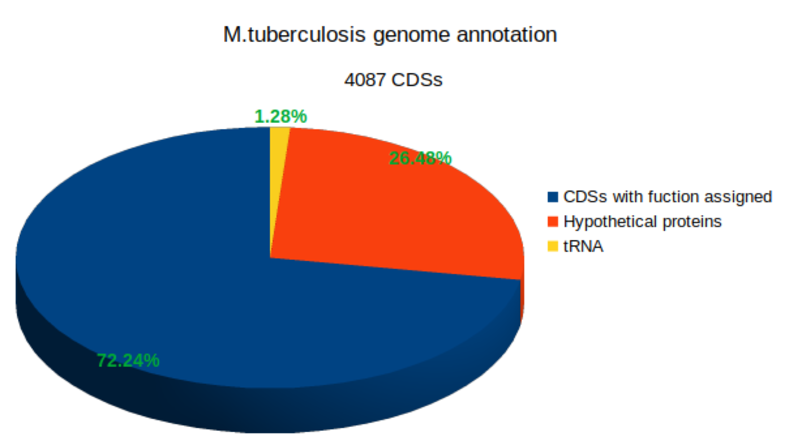

Supplement: S2 Fig — Average of CDSs annotated by gene prediction and homology of sequence, 2,880 CDSs (72.24) had a functional assignment in the annotation, 1,122(26.48%) corresponded to hypothetical proteins, and 52 (1.28) to tRNA. (TIF) [file pone.0304060.s006.tif]

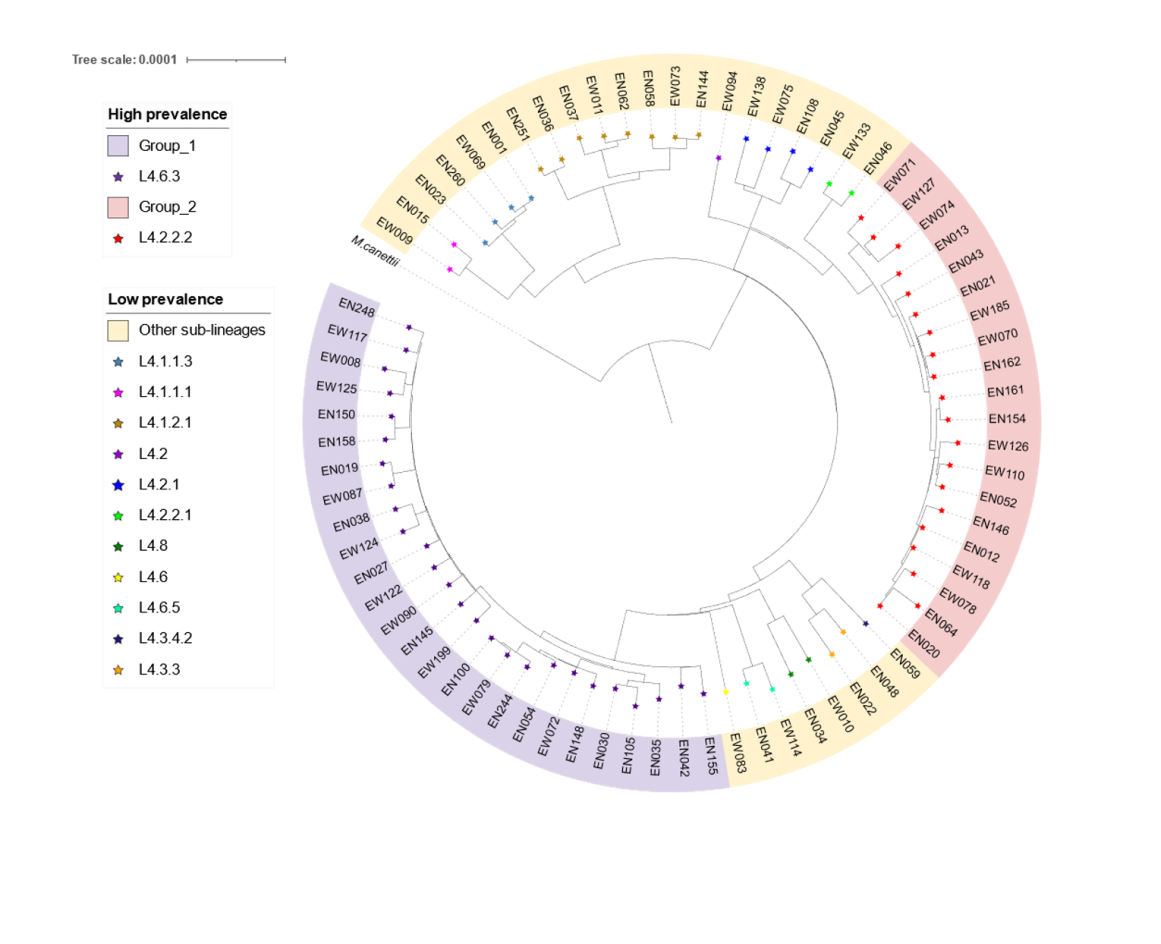

Supplement: S3 Fig — The outgroup was M. canetti CIPT 140010059 and the shapes on each tip of all branches indicate sub-lineages of L4. The red shades correspond to isolates with a high prevalence of L4.2.2.2 sub-lineage, the light purple corresponds high prevalence of L4.6.3, and the yellow shades correspond to all isolates with a low prevalence in western Ethiopia. (TIF) [file pone.0304060.s007.tif]

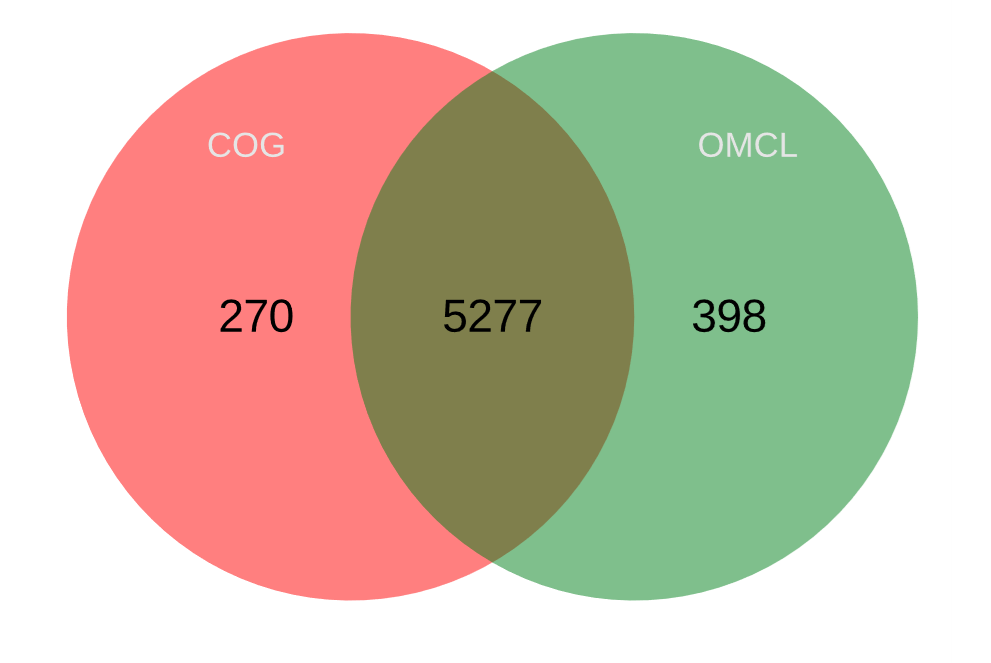

Supplement: S4 Fig — The intersection of COG and OMCL algorithms is the total number of gene clusters of which it is composed of the set 75 genomes. (TIF) [file pone.0304060.s008.tif]

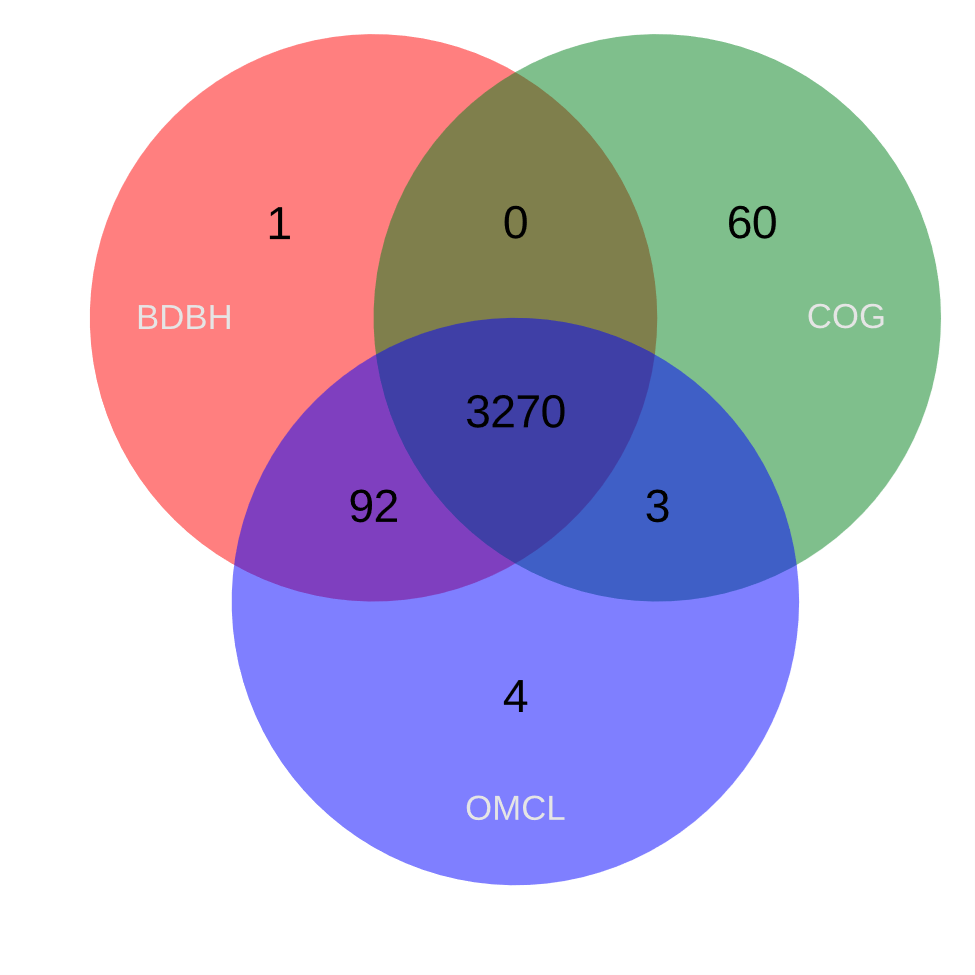

Supplement: S5 Fig — The intersection of three algorithms of the cluster of orthologous genes. In the center, the number of clusters shared in 100% of the isolates. Unique clusters were identified by BDBH (1), OMLC (4), and COG (60) algorithms. Some gene clusters are observed shared between two of the three algorithms (script comare_clusters.pl from get_homologues). (TIF) [file pone.0304060.s009.tif]

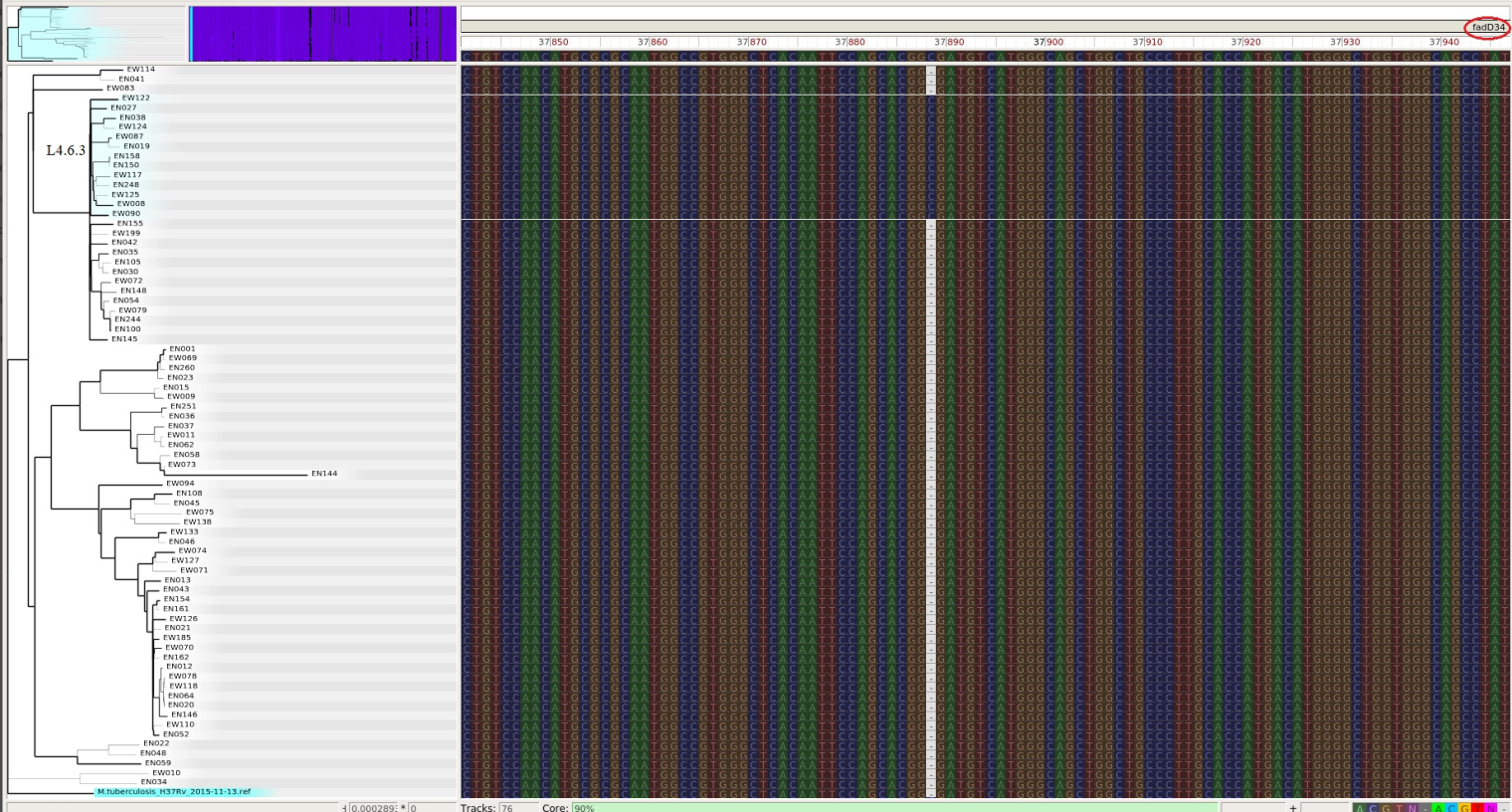

Supplement: S6 Fig — The left side shows the core phylogeny of 75 isolates using H37Rv as a reference (GenBank accession number NC_000962.3). The right side corresponds to a multi-genomic alignment against the phylogenetic tree. The red letter (A) in the white box shows the variant (SNP) in the fadD34 gene of high prevalence L4.6.3 genomes. (TIF) [file pone.0304060.s010.tif]

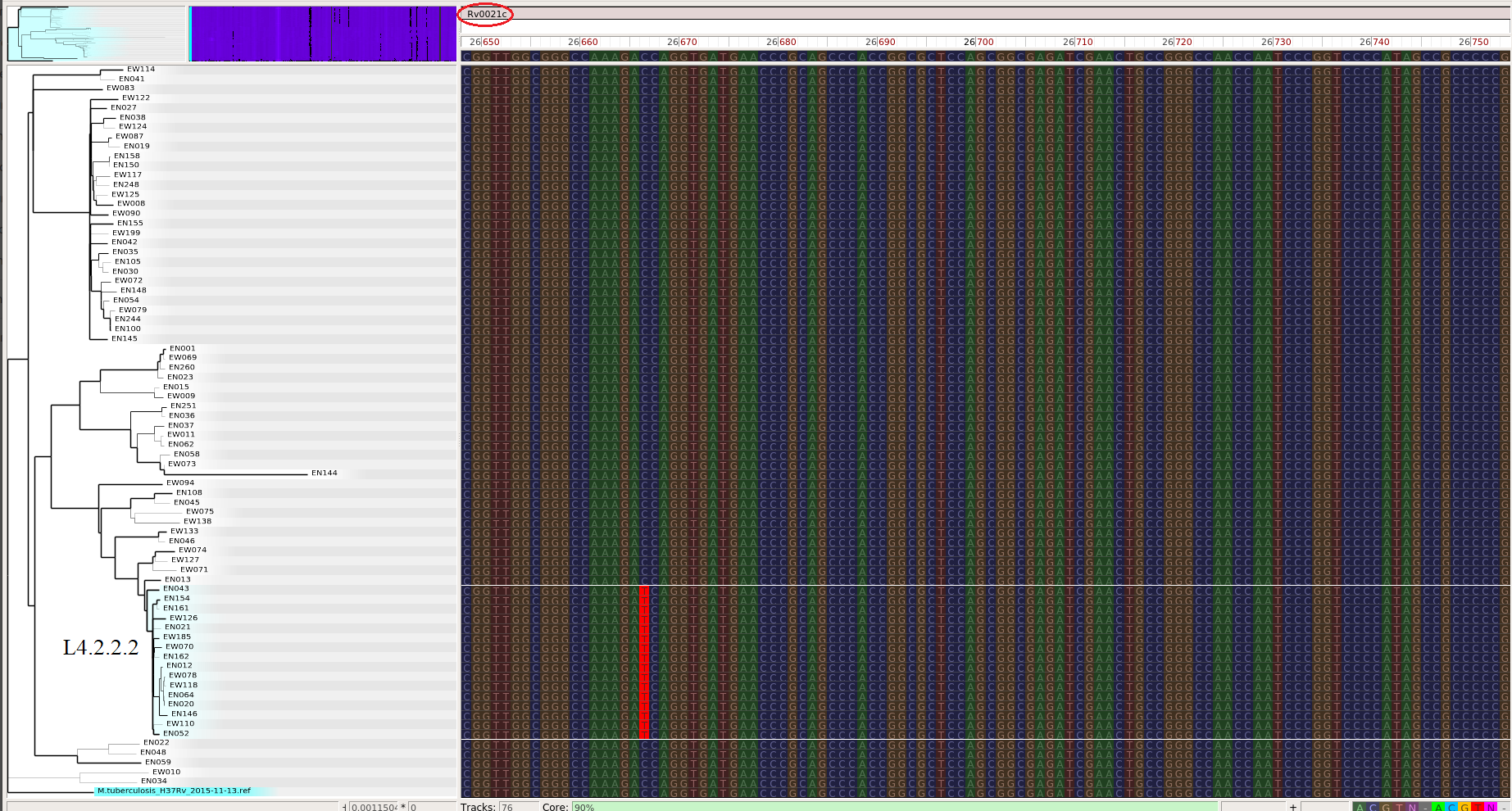

Supplement: S7 Fig — The left side shows the core phylogeny of 75 isolates using H37Rv as a reference (GenBank accession number NC_000962.3). The right side corresponds to a multi-genome alignment against the phylogenetic tree. The red letter (A) in the white box shows the variant (SNP) in the Rv0021c gene of high prevalence L4.2.2.2 genomes. (TIF) [file pone.0304060.s011.tif]

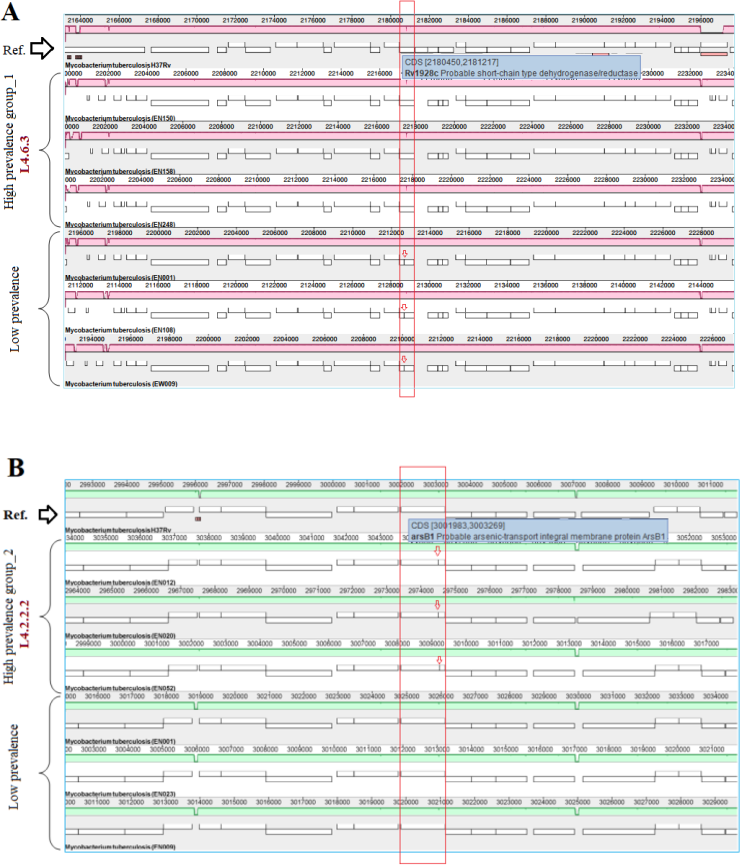

Supplement: S8 Fig — The joined segmented rectangles or squares correspond to the CDS annotated. The blue-shaded rectangle shows the locus annotation for the Rv1928c (A) and arsB1 (B) genes in H37Rv, reference genome (GenBank accession number NC_000962.3). (A) The red arrow in the red box shows the variant difference of the Rv1928c gene between high prevalence L4.6.3 genomes with a complete CDS and low prevalence with two smaller CDS are observed, the first with a premature stop codon due to deletion and the second after the deletion despite having a start codon possibly not be functional. (B) The red arrow in the red box shows the genetic variant difference of the arsB1 gene between low prevalence genomes with a complete CDS and high prevalence L4.2.2.2 with two smaller CDS are observed, the first with a premature stop codon due to deletion and the second after the deletion despite having a start codon possibly not be functional. (TIF) [file pone.0304060.s012.tif]

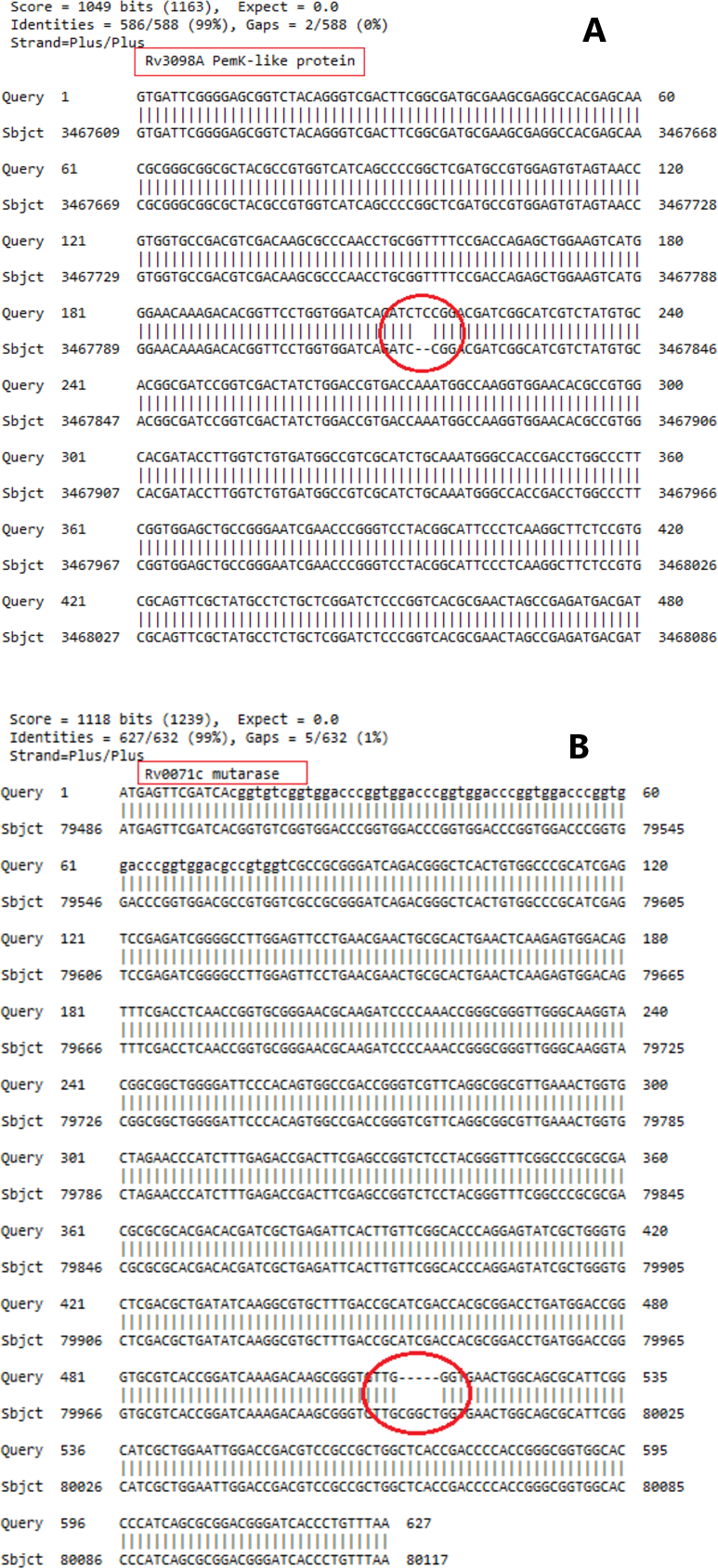

Supplement: S9 Fig — The gap in the red circle shows the insertion in the Rv3098A (A) and deletion in the Rv0071c (B) genes of high prevalence L4.6.3 genomes. (TIF) [file pone.0304060.s013.tif]
