# Supplementary material for: Pangenome and genomic signatures linked to the dominance of the lineage-4 of Mycobacterium tuberculosis isolated from extrapulmonary tuberculosis patients in western Ethiopia
Source: PLoS One. 2024 Jul 25;19(7):e0304060. doi: 10.1371/journal.pone.0304060 (PMC11271921; doi:10.1371/journal.pone.0304060)
Supplement: S1 File — (DOCX) [file pone.0304060.s014.docx]

Pangenome and genomic signatures linked to the dominance of the lineage-4 of *Mycobacterium tuberculosis* isolated from extrapulmonary tuberculosis patients in western Ethiopia

Basha Chekesa^1,2*^, Harinder Singh^3^, Norberto Gonzalez-Juarbe^3^, Sanjay Vashee^3^, Rosana Wiscovitch-Russo^3^, Christopher L. Dupont^4^, Musse Girma^1^, Oudessa Kerro^5^, Balako Gumi^1^, Gobena Ameni^1,6*^.

Corresponding author’s emails: [balchachekesa@gmail.com](mailto:balchachekesa@gmail.com) (B.C) and [gobena.ameni@uaeu.ac.ae](mailto:gobena.ameni@uaeu.ac.ae) (G.A)

**S1 Table. Patient characteristics, isolates prevalence category and sequencing read features (N=75)**

| No. | ID | Age | Sex | Prevalence | Total reads | % Mapped reads | Coverage mean | Total bases | %Total bases |
| --- | --- | --- | --- | --- | --- | --- | --- | --- | --- |
| 1 | EN001 | 65 | F | Low | 8116305 | 99.48 | 237.61 | 4398015 | 1 |
| 2 | EN012 | 18 | M | L4.2.2.2 | 4349942 | 96.41 | 121.29 | 4390415 | 1 |
| 3 | EN013 | 65 | M | L4.2.2.2 | 7385239 | 98.69 | 213.73 | 4391993 | 1 |
| 4 | EN015 | 12 | M | Low | 35649021 | 98.9 | 1032.33 | 4401365 | 1 |
| 5 | EN019 | 32 | F | L4.6.3 | 25828349 | 99.69 | 763.76 | 4405040 | 1 |
| 6 | EN020 | 45 | M | L4.2.2.2 | 6802589 | 69.97 | 142.72 | 4388414 | 0.99 |
| 7 | EN021 | 12 | M | L4.2.2.2 | 8506475 | 99.42 | 252.83 | 4391965 | 1 |
| 8 | EN022 | 30 | F | Low | 7038627 | 99.74 | 209.77 | 4367695 | 0.99 |
| 9 | EN023 | 9 | M | Low | 6724012 | 99.5 | 197.22 | 4397282 | 1 |
| 10 | EN027 | 18 | F | L4.6.3 | 8004594 | 91.92 | 212.68 | 4401172 | 1 |
| 11 | EN030 | 21 | F | L4.6.3 | 5606616 | 98.23 | 159.6 | 4402357 | 1 |
| 12 | EN034 | 15 | F | Low | 18676028 | 97.67 | 540.75 | 4400642 | 1 |
| 13 | EN035 | 45 | F | L4.6.3 | 7538891 | 99.09 | 218.22 | 4401063 | 1 |
| 14 | EN036 | 60 | F | Low | 10172739 | 99.14 | 291.5 | 4396314 | 1 |
| 15 | EN037 | 27 | F | Low | 8389876 | 98.73 | 247.01 | 4393361 | 1 |
| 16 | EN038 | 27 | F | L4.6.3 | 8281340 | 99.05 | 232.6 | 4403080 | 1 |
| 17 | EN041 | 25 | M | Low | 7047354 | 99.62 | 201.62 | 4408366 | 1 |
| 18 | EN042 | 18 | F | L4.6.3 | 9231478 | 99.58 | 263.59 | 4404124 | 1 |
| 19 | EN043 | 30 | M | L4.2.2.2 | 6603590 | 99.33 | 193.17 | 4387973 | 0.99 |
| 20 | EN045 | 48 | M | Low | 14169589 | 83.82 | 339.97 | 4398971 | 1 |
| 21 | EN046 | 25 | F | Low | 6532229 | 62.49 | 116.58 | 4396037 | 1 |
| 22 | EN048 | 15 | F | Low | 4152284 | 98.13 | 117.67 | 4389736 | 1 |
| 23 | EN052 | 15 | F | L4.2.2.2 | 6789102 | 99.28 | 199.75 | 4390154 | 1 |
| 24 | EN054 | 32 | M | L4.6.3 | 8744575 | 99.63 | 253.59 | 4400949 | 1 |
| 25 | EN058 | 21 | F | Low | 9443990 | 92.01 | 254.79 | 4390284 | 1 |
| 26 | EN059 | 22 | F | Low | 9220887 | 99.78 | 270.42 | 4357257 | 0.99 |
| 27 | EN062 | 47 | F | Low | 9083716 | 99.49 | 266.56 | 4394530 | 1 |
| 28 | EN064 | 35 | M | L4.2.2.2 | 8340493 | 99.46 | 242.99 | 4391527 | 1 |
| 29 | EN100 | NA | M | L4.6.3 | 2258206 | 99.73 | 63.38 | 4397033 | 1 |
| 30 | EN105 | 17 | M | L4.6.3 | 1322877 | 99.45 | 36.32 | 4399584 | 1 |
| 31 | EN108 | NA | F | Low | 1607458 | 99.2 | 44.1 | 4390279 | 1 |
| 32 | EN144 | 33 | M | Low | 1162067 | 84.5 | 26.79 | 4389104 | 0.99 |
| 33 | EN145 | 51 | F | L4.6.3 | 1285045 | 99.71 | 35.54 | 4393011 | 1 |
| 34 | EN146 | 26 | F | L4.2.2.2 | 1566468 | 99.28 | 42.77 | 4383835 | 0.99 |
| 35 | EN148 | 20 | F | L4.6.3 | 9438563 | 68.58 | 180.72 | 4405531 | 1 |
| 36 | EN150 | 31 | M | L4.6.3 | 20106500 | 99.37 | 563.42 | 4404392 | 1 |
| 37 | EN154 | 45 | M | L4.2.2.2 | 9100210 | 99.44 | 250.19 | 4393151 | 1 |
| 38 | EN155 | 29 | M | L4.6.3 | 1013758 | 99.56 | 28.43 | 4383657 | 0.99 |
| 39 | EN158 | 18 | F | L4.6.3 | 2329835 | 99.6 | 66.15 | 4396839 | 1 |
| 40 | EN161 | 28 | M | L4.2.2.2 | 4233128 | 99.36 | 116.49 | 4390648 | 1 |
| 41 | EN162 | 52 | M | L4.2.2.2 | 2903662 | 99.08 | 78.94 | 4388039 | 0.99 |
| 42 | EN244 | 43 | F | L4.6.3 | 7046065 | 97.43 | 191.89 | 4405743 | 1 |
| 43 | EN248 | 18 | F | L4.6.3 | 3254972 | 98.31 | 88.99 | 4400025 | 1 |
| 44 | EN251 | 17 | F | Low | 1309692 | 99.47 | 35.86 | 4389422 | 0.99 |
| 45 | EN260 | 27 | F | Low | 1530074 | 99.46 | 42.72 | 4394234 | 1 |
| 46 | EW008 | 18 | F | L4.6.3 | 6282292 | 77.64 | 142.56 | 4398394 | 1 |
| 47 | EW009 | 20 | M | Low | 7871616 | 98.78 | 225.64 | 4399417 | 1 |
| 48 | EW010 | 21 | F | Low | 5937751 | 78.33 | 135.83 | 4391710 | 1 |
| 49 | EW011 | 26 | F | Low | 6436282 | 99.19 | 185.45 | 4394208 | 1 |
| 50 | EW069 | 25 | M | Low | 2784627 | 99.53 | 77.37 | 4396096 | 1 |
| 51 | EW070 | 2 | M | L4.2.2.2 | 3120925 | 99.1 | 88.76 | 4386423 | 0.99 |
| 52 | EW071 | 30 | F | L4.2.2.2 | 781787 | 99.4 | 21.77 | 4379774 | 0.99 |
| 53 | EW072 | 26 | M | L4.6.3 | 1143230 | 99.74 | 32.63 | 4393711 | 1 |
| 54 | EW073 | 35 | M | Low | 1477413 | 99.56 | 40.92 | 4386707 | 0.99 |
| 55 | EW074 | 50 | M | L4.2.2.2 | 900016 | 99.42 | 25.62 | 4385769 | 0.99 |
| 56 | EW075 | 24 | F | Low | 1995478 | 99.4 | 56.51 | 4391816 | 1 |
| 57 | EW078 | 48 | F | L4.2.2.2 | 1821411 | 99.42 | 50.91 | 4385499 | 0.99 |
| 58 | EW079 | 45 | M | L4.6.3 | 862587 | 99.74 | 23.93 | 4395951 | 1 |
| 59 | EW083 | 65 | M | Low | 3727657 | 99.52 | 103.42 | 4403330 | 1 |
| 60 | EW087 | 15 | F | L4.6.3 | 4424555 | 99.73 | 124.47 | 4398777 | 1 |
| 61 | EW090 | 19 | F | L4.6.3 | 1500162 | 99.33 | 41.75 | 4395009 | 1 |
| 62 | EW094 | 26 | F | Low | 4084790 | 99.43 | 116.67 | 4398011 | 1 |
| 63 | EW110 | NA | NA | L4.2.2.2 | 833098 | 99.25 | 23.36 | 4375381 | 0.99 |
| 64 | EW114 | 18 | F | Low | 1051504 | 99.52 | 28.81 | 4382909 | 0.99 |
| 65 | EW117 | NA | NA | L4.6.3 | 1721966 | 99.59 | 48.24 | 4386702 | 0.99 |
| 66 | EW118 | 35 | F | L4.2.2.2 | 1939649 | 99.3 | 53.95 | 4388022 | 0.99 |
| 67 | EW122 | 23 | M | L4.6.3 | 1754680 | 91.25 | 43.83 | 4400200 | 1 |
| 68 | EW124 | 46 | M | L4.6.3 | 849313 | 99.51 | 23.73 | 4393687 | 1 |
| 69 | EW125 | 27 | M | L4.6.3 | 2203826 | 99.64 | 61.89 | 4401167 | 1 |
| 70 | EW126 | NA | NA | L4.2.2.2 | 1527611 | 99.44 | 41.13 | 4386455 | 0.99 |
| 71 | EW127 | 18 | M | L4.2.2.2 | 1416113 | 98.82 | 38.78 | 4396618 | 1 |
| 72 | EW133 | 50 | M | Low | 1989244 | 98.6 | 56.67 | 4393598 | 1 |
| 73 | EW138 | 13 | M | Low | 1832848 | 99.28 | 49.58 | 4403677 | 1 |
| 74 | EW185 | 25 | M | L4.2.2.2 | 3361815 | 99.45 | 91.54 | 4389275 | 0.99 |
| 75 | EW199 | 30 | F | L4.6.3 | 2207063 | 96.71 | 60.07 | 4402657 | 1 |
| Average | |  |  |  | **5676530.92** | **96.632** | **157.3708** | **4393674.11** | **0.9973** |

*NA* not available, *Low* isolates categorized in the low prevalent group

**S2 Table. Scaffold’s features, annotated genes, and quality of the genome assembly (N=75)**

| No. | ID | N50(bp) | Largest contig | No. contigs | Total length | Genome fraction (%) | G+C_% | CDS | tRNA | Compl  eteness | Contam  ination | Strain  Hetero  geneity |
| --- | --- | --- | --- | --- | --- | --- | --- | --- | --- | --- | --- | --- |
| 1 | EN001 | 83307 | 228341 | 112 | 4356767 | 97.74 | 65.5 | 4054 | 53 | 100 | 0 | 0 |
| 2 | EN012 | 65893 | 230068 | 125 | 4354664 | 97.65 | 65.4 | 4085 | 52 | 100 | 0 | 0 |
| 3 | EN013 | 64599 | 229989 | 108 | 4368835 | 97.9 | 65.4 | 4058 | 57 | 100 | 0 | 0 |
| 4 | EN015 | 80705 | 213145 | 101 | 4404978 | 97.92 | 65.4 | 4093 | 55 | 100 | 0 | 0 |
| 5 | EN019 | 84352 | 222753 | 102 | 4356067 | 98.16 | 65.4 | 4078 | 53 | 100 | 0 | 0 |
| 6 | EN020 | 7232 | 230051 | 124 | 4353495 | 97.73 | 65.4 | 4059 | 53 | 100 | 0 | 0 |
| 7 | EN021 | 82712 | 230030 | 104 | 4352661 | 97.77 | 65.5 | 4057 | 53 | 100 | 0 | 0 |
| 8 | EN022 | 79191 | 202329 | 114 | 4319615 | 97.32 | 65.4 | 4040 | 53 | 100 | 0 | 0 |
| 9 | EN023 | 82284 | 228390 | 116 | 4358141 | 97.77 | 65.5 | 4066 | 53 | 100 | 0 | 0 |
| 10 | EN027 | 19688 | 222619 | 122 | 4359640 | 97.91 | 65.5 | 4073 | 53 | 100 | 0 | 0 |
| 11 | EN030 | 71018 | 222452 | 123 | 4360087 | 97.97 | 65.4 | 4069 | 53 | 100 | 0 | 0 |
| 12 | EN034 | 64021 | 226585 | 105 | 4340499 | 97.73 | 65.4 | 4072 | 53 | 100 | 0 | 0 |
| 13 | EN035 | 84001 | 222660 | 112 | 4351428 | 97.94 | 65.5 | 4069 | 53 | 100 | 0 | 0 |
| 14 | EN036 | 59426 | 228073 | 139 | 4372516 | 97.85 | 65.4 | 4096 | 53 | 100 | 0 | 0 |
| 15 | EN037 | 84089 | 223401 | 105 | 4360389 | 97.91 | 65.5 | 4070 | 53 | 100 | 0 | 0 |
| 16 | EN038 | 80914 | 197658 | 118 | 4350800 | 97.99 | 65.5 | 4058 | 53 | 100 | 0 | 0 |
| 17 | EN041 | 69543 | 222814 | 130 | 4389165 | 97.97 | 65.4 | 4137 | 53 | 100 | 0 | 0 |
| 18 | EN042 | 81489 | 192413 | 124 | 4350570 | 97.83 | 65.4 | 4090 | 53 | 100 | 0 | 0 |
| 19 | EN043 | 84476 | 230244 | 113 | 4358807 | 97.83 | 65.4 | 4083 | 53 | 100 | 0 | 0 |
| 20 | EN045 | 52390 | 205112 | 111 | 4369379 | 97.84 | 65.4 | 4086 | 52 | 100 | 0 | 0 |
| 21 | EN046 | 22537 | 226175 | 147 | 4373395 | 97.77 | 65.4 | 4102 | 53 | 100 | 0 | 0 |
| 22 | EN048 | 82262 | 222774 | 130 | 4332631 | 97.62 | 65.5 | 4059 | 53 | 100 | 0 | 0 |
| 23 | EN052 | 79227 | 229977 | 114 | 4355143 | 97.74 | 65.4 | 4064 | 52 | 100 | 0 | 0 |
| 24 | EN054 | 71197 | 222699 | 122 | 4351620 | 97.72 | 65.4 | 4096 | 53 | 100 | 0 | 0 |
| 25 | EN058 | 26700 | 159455 | 128 | 4354955 | 97.63 | 65.5 | 4063 | 53 | 100 | 0 | 0 |
| 26 | EN059 | 97788 | 223066 | 109 | 4302144 | 96.8 | 65.4 | 4036 | 52 | 100 | 0 | 0 |
| 27 | EN062 | 72379 | 223495 | 117 | 4353111 | 97.79 | 65.4 | 4076 | 53 | 100 | 0 | 0 |
| 28 | EN064 | 83858 | 230253 | 110 | 4358996 | 97.74 | 65.5 | 4061 | 53 | 100 | 0 | 0 |
| 29 | EN100 | 65782 | 222503 | 164 | 4335943 | 97.39 | 65.4 | 4100 | 52 | 100 | 0 | 0 |
| 30 | EN105 | 65752 | 188846 | 144 | 4332808 | 97.48 | 65.4 | 4052 | 52 | 100 | 0 | 0 |
| 31 | EN108 | 56290 | 178896 | 175 | 4333064 | 97.1 | 65.4 | 4150 | 52 | 100 | 0 | 0 |
| 32 | EN144 | 1185 | 158462 | 326 | 4408724 | 96.91 | 65.5 | 4173 | 53 | 100 | 2 | 0 |
| 33 | EN145 | 64158 | 158665 | 158 | 4330700 | 97.35 | 65.4 | 4091 | 53 | 100 | 0 | 0 |
| 34 | EN146 | 60826 | 230194 | 158 | 4337763 | 97.18 | 65.4 | 4110 | 52 | 97.67 | 0 | 0 |
| 35 | EN148 | 38258 | 222837 | 137 | 4401984 | 97.98 | 65.5 | 4113 | 56 | 100 | 0 | 0 |
| 36 | EN150 | 80970 | 203497 | 97 | 4360371 | 98.13 | 65.5 | 4053 | 57 | 100 | 0 | 0 |
| 37 | EN154 | 123769 | 207003 | 89 | 4365277 | 97.98 | 65.5 | 4044 | 53 | 100 | 0 | 0 |
| 38 | EN155 | 60507 | 193975 | 171 | 4305674 | 96.82 | 65.4 | 4051 | 53 | 100 | 0 | 0 |
| 39 | EN158 | 67989 | 157173 | 143 | 4334818 | 97.61 | 65.4 | 4104 | 52 | 100 | 0 | 0 |
| 40 | EN161 | 83851 | 230181 | 104 | 4358711 | 97.74 | 65.4 | 4085 | 53 | 100 | 0 | 0 |
| 41 | EN162 | 66116 | 230046 | 133 | 4354043 | 97.52 | 65.4 | 4086 | 53 | 100 | 0 | 0 |
| 42 | EN244 | 32504 | 222737 | 100 | 4360225 | 98 | 65.4 | 4074 | 53 | 100 | 0 | 0 |
| 43 | EN248 | 51670 | 162471 | 119 | 4358597 | 97.86 | 65.5 | 4103 | 55 | 100 | 0 | 0 |
| 44 | EN251 | 64161 | 228335 | 150 | 4337058 | 97.2 | 65.5 | 4079 | 53 | 100 | 0 | 0 |
| 45 | EN260 | 17055 | 224795 | 151 | 4343548 | 97.51 | 65.4 | 4122 | 54 | 100 | 0 | 0 |
| 46 | EW008 | 36191 | 222802 | 142 | 4344117 | 97.67 | 65.5 | 4081 | 53 | 100 | 0 | 0 |
| 47 | EW009 | 71028 | 193618 | 121 | 4358922 | 97.78 | 65.5 | 4077 | 54 | 100 | 0 | 0 |
| 48 | EW010 | 31976 | 226279 | 139 | 4351044 | 97.51 | 65.5 | 4100 | 53 | 100 | 0 | 0 |
| 49 | EW011 | 67990 | 223490 | 107 | 4360564 | 97.79 | 65.4 | 4085 | 54 | 100 | 0 | 0 |
| 50 | EW069 | 81381 | 228321 | 127 | 4352518 | 97.62 | 65.5 | 4074 | 53 | 100 | 0 | 0 |
| 51 | EW070 | 64186 | 184065 | 137 | 4345021 | 97.42 | 65.4 | 4074 | 53 | 100 | 0 | 0 |
| 52 | EW071 | 55894 | 227955 | 178 | 4332499 | 96.87 | 65.3 | 4121 | 53 | 100 | 0 | 0 |
| 53 | EW072 | 51636 | 216436 | 174 | 4326399 | 97.15 | 65.4 | 4080 | 53 | 100 | 0 | 0 |
| 54 | EW073 | 63593 | 155526 | 158 | 4343573 | 97.3 | 65.4 | 4122 | 53 | 100 | 0 | 0 |
| 55 | EW074 | 47687 | 197688 | 188 | 4333427 | 97.06 | 65.4 | 4117 | 53 | 100 | 0 | 0 |
| 56 | EW075 | 64600 | 202907 | 146 | 4337738 | 97.27 | 65.4 | 4078 | 52 | 100 | 0 | 0 |
| 57 | EW078 | 61982 | 230038 | 141 | 4340503 | 97.37 | 65.4 | 4089 | 53 | 100 | 0 | 0 |
| 58 | EW079 | 57841 | 141048 | 182 | 4322876 | 97.22 | 65.4 | 4118 | 52 | 100 | 0 | 0 |
| 59 | EW083 | 79304 | 222909 | 127 | 4358922 | 97.94 | 65.5 | 4101 | 53 | 100 | 0 | 0 |
| 60 | EW087 | 67989 | 222697 | 134 | 4345619 | 97.77 | 65.5 | 4076 | 53 | 100 | 0 | 0 |
| 61 | EW090 | 64197 | 141040 | 159 | 4340665 | 97.46 | 65.4 | 4093 | 53 | 100 | 0 | 0 |
| 62 | EW094 | 79287 | 229941 | 115 | 4362760 | 97.85 | 65.4 | 4122 | 53 | 100 | 0 | 0 |
| 63 | EW110 | 49661 | 230016 | 179 | 4327389 | 97.01 | 65.4 | 4104 | 53 | 100 | 0 | 0 |
| 64 | EW114 | 55055 | 158768 | 169 | 4325169 | 96.95 | 65.4 | 4081 | 53 | 100 | 0 | 0 |
| 65 | EW117 | 63794 | 156671 | 157 | 4332898 | 97.42 | 65.4 | 4086 | 54 | 100 | 0 | 0 |
| 66 | EW118 | 70926 | 230197 | 134 | 4344886 | 97.35 | 65.4 | 4092 | 53 | 100 | 0 | 0 |
| 67 | EW122 | 43755 | 154303 | 157 | 4353498 | 97.58 | 65.4 | 4091 | 53 | 100 | 0 | 0 |
| 68 | EW124 | 47826 | 141045 | 194 | 4319618 | 97.09 | 65.4 | 4092 | 53 | 100 | 0 | 0 |
| 69 | EW125 | 71022 | 222655 | 136 | 4351919 | 97.72 | 65.4 | 4108 | 53 | 100 | 0 | 0 |
| 70 | EW126 | 58980 | 179539 | 152 | 4347403 | 97.43 | 65.4 | 4100 | 53 | 100 | 0 | 0 |
| 71 | EW127 | 59835 | 166616 | 138 | 4363369 | 97.7 | 65.4 | 4111 | 52 | 100 | 0 | 0 |
| 72 | EW133 | 64161 | 230131 | 153 | 4366664 | 97.58 | 65.4 | 4129 | 53 | 100 | 0 | 0 |
| 73 | EW138 | 64118 | 158447 | 153 | 4359054 | 97.73 | 65.4 | 4140 | 53 | 100 | 0 | 0 |
| 74 | EW185 | 78989 | 229991 | 126 | 4359363 | 97.68 | 65.4 | 4082 | 53 | 100 | 0 | 0 |
| 75 | EW199 | 47223 | 175936 | 138 | 4342724 | 97.66 | 65.5 | 4065 | 55 | 100 | 0 | 0 |
| Average | | **63203** | **206249** | **136.87** | **4350412** | **97.59** | **65.43** | **4087** | **53.12** | **99.97** | **0.027** | **0** |

**S3 Table. Mutations associated with** **L4.6.3 (N=26) or low prevalence (N=29) of MTB lineage-4 in western Ethiopia.**

| **Gene** | **#Position** | **Variants or gene deletion** | **Genomes number high prev. N = 26** | **Genomes number low prev. N = 29** | **Benjamini-H. adjusted P-value** | **Mutations association** |
| --- | --- | --- | --- | --- | --- | --- |
| ***Rv0071*** | 79999-80003 | g.514-518delCGGCT | 26 | 0 | 7.22E-14 | HP |
| ***vapC28/Rv0609*** | - | - | 26 | 0 | 7.22E-14 | HP |
| ***Rv3098A*** | 3467819 | g.214-215insTC | 26 | 0 | 7.22E-14 | HP |
| ***icl2*** | - | - | 26 | 8 | 3.58E-07 | HP |
| ***Rv1928c*** | 2180818 | g.400delT | 0 | 21 | 3.58E-07 | LP |
| ***PE_PGRS20*** | 1191497 | p.Thr218Ser (acg/Tcg) | 0 | 18 | 1.46E-05 | LP |
| ***Rv3093c*** | 3462145 | g.619-620insGGCGC | 0 | 16 | 0.0001 | LP |
| ***fadD34*** | 37887 | g.629-630insC | 13 | 0 | 0.0003 | HP |
| ***PE_PGRS6/Rv0532*** | 624078 | p.Ala429Gly(gcg/gGg) | 13 | 0 | 0.0003 | HP |
|  | 624089 | p.Asn433Asp (aac/Gac) |  |  |  |  |
| ***Rv0025*** | 29483 | g.239delA | 0 | 14 | 0.0007 | LP |
| ***Rv2994*** | 3352078 | p.Trp270_ (tgg/tgA) | 10 | 0 | 0.005 | HP |

*HP* high prevalence, *LP* low prevalence

| **Gene** | **#Position** | **Variants/gene deletion** | **Genomes number high prev. N = 20** | **Genomes number low prev. N = 29** | **Benjamini-H. adjusted P-value** | **Mutations association** |
| --- | --- | --- | --- | --- | --- | --- |
| ***Rv2717c*** | 3030197-3030213 | g.148-165del | 20 | 2 | 1.68E-09 | HP |
|  | 3030214 | p.Thr50Ser (acg/Tcg) |  |  |  |  |
| ***Rv0021c*** | 26666 | p.Trp72_ (tgg/tgA) | 16 | 0 | 1.99E-07 | HP |
| ***NarX/ Rv1736c*** | 1963855 | p.Trp111_ (tgg/tAg) | 15 | 0 | 9.004E-07 | HP |
| ***arsB1/Rv2685*** | 3003085 | g.1104delC | 20 | 6 | 1.42E-06 | HP |
| ***Rv0654*** | 751297 | g.1299_3000insC | 20 | 7 | 1.59E-06 | HP |
| ***Rv0075*** | 84831 | g.836delC | 20 | 7 | 1.59E-06 | HP |
| ***Rv0073*** | 82438 | g.763delT | 20 | 7 | 1.59E-06 | HP |
| ***Rv1132*** | 1257826 | g.502delG | 20 | 7 | 1.59E-06 | HP |
| ***eccC4*** | 3867404 | p.Glu279_ (gag/Tag) | 20 | 7 | 1.59E-06 | HP |
|  | 3867973 | p.His89Arg(cac/cGc) | 20 | 0 | 1.01E-11 |  |
| ***Rv3047c*** | 3408302 | g.3delC | 20 | 7 | 1.59E-06 | HP |
| ***TB27.3/ Rv0577*** | 671406 | g.242-243insTC | 20 | 7 | 1.59E-06 | HP |
| ***PE_PGRS42*** | 2796214 | g.1172-1173insC | 20 | 8 | 1.85E-05 | HP |
| ***Rv0025*** | 29483 | g.239delA | 0 | 14 | 0.004 | LP |

**S4 Table. Mutations associated with L4.2.2.2 (N=20) or low prevalence (N=29) of MTB lineage-4 in western Ethiopia.**

*HP* high prevalence; *LP* low prevalence

**S1 Fig. Prevalence of MTB sublineage-4 in western Ethiopia.** A histogram denoting the distribution of MTB L4 sublineages and the numbers on top of the bars indicate the percentage of sub-lineages. Except for L4.6.3 and L4.2.2.2, all other sub-lineages of L4 were classified as low prevalent groups. *L* lineage.


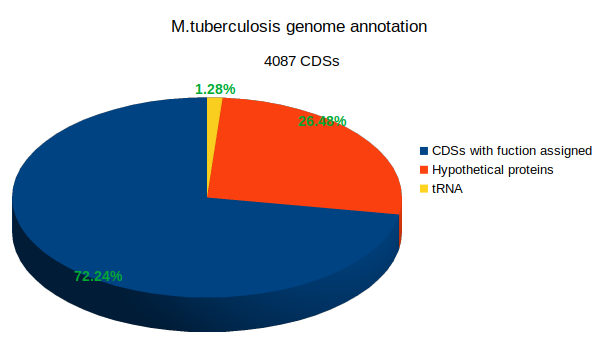


**S2 Fig.** **Global annotation of the 75 MTB genome.** Average of CDSs annotated by gene prediction and homology of sequence, 2,880 CDSs (72.24) had a functional assignment in the annotation, 1,122(26.48%) corresponded to hypothetical proteins, and 52 (1.28) to tRNA.


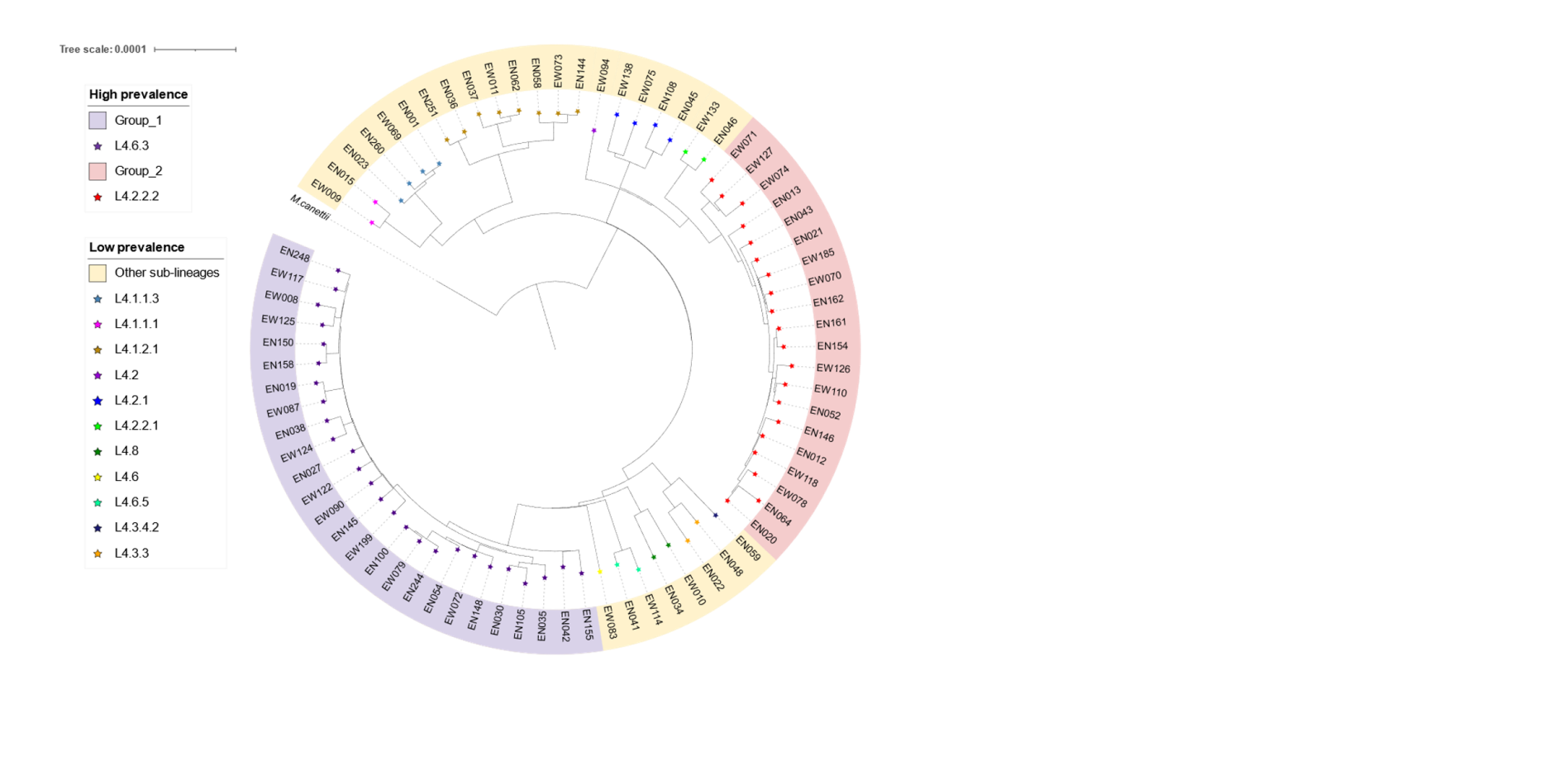
 **S3 Fig**. **Whole genome multilocus sequence typing (wgMLST) based phylogeny.** The outgroup was *M. canetti* CIPT 140010059 and the shapes on each tip of all branches indicate sub-lineages of L4. The red shades correspond to isolates with a high prevalence of L4.2.2.2 sub-lineage, the light purple corresponds high prevalence of L4.6.3, and the yellow shades correspond to all isolates with a low prevalence in western Ethiopia.


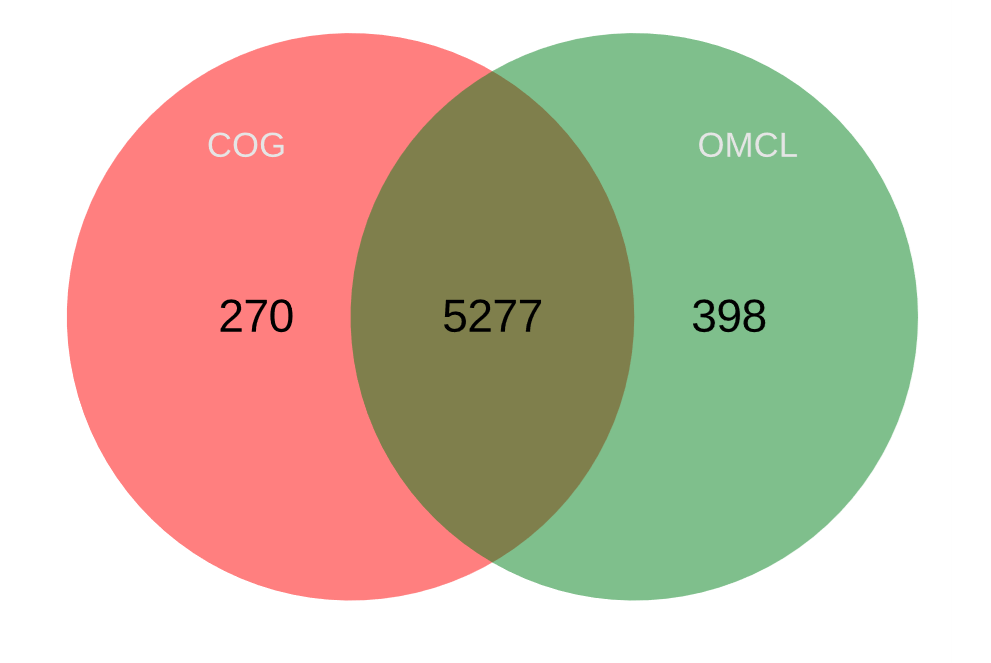
 **S4 Fig. Pangeneome of MTB Lineage-4.** The intersection of COG and OMCL algorithms is the total number of gene clusters of which it is composed of the set 75 genomes.

**S5 Fig. Core Genome of MTB Lineage 4.** The intersection of three algorithms of the cluster of orthologous genes. In the center, the number of clusters shared in 100% of the isolates. Unique clusters were identified by BDBH (1), OMLC (4), and COG (60) algorithms. Some gene clusters are observed shared between two of the three algorithms (script *comare_clusters.pl* from get_homologues).

**
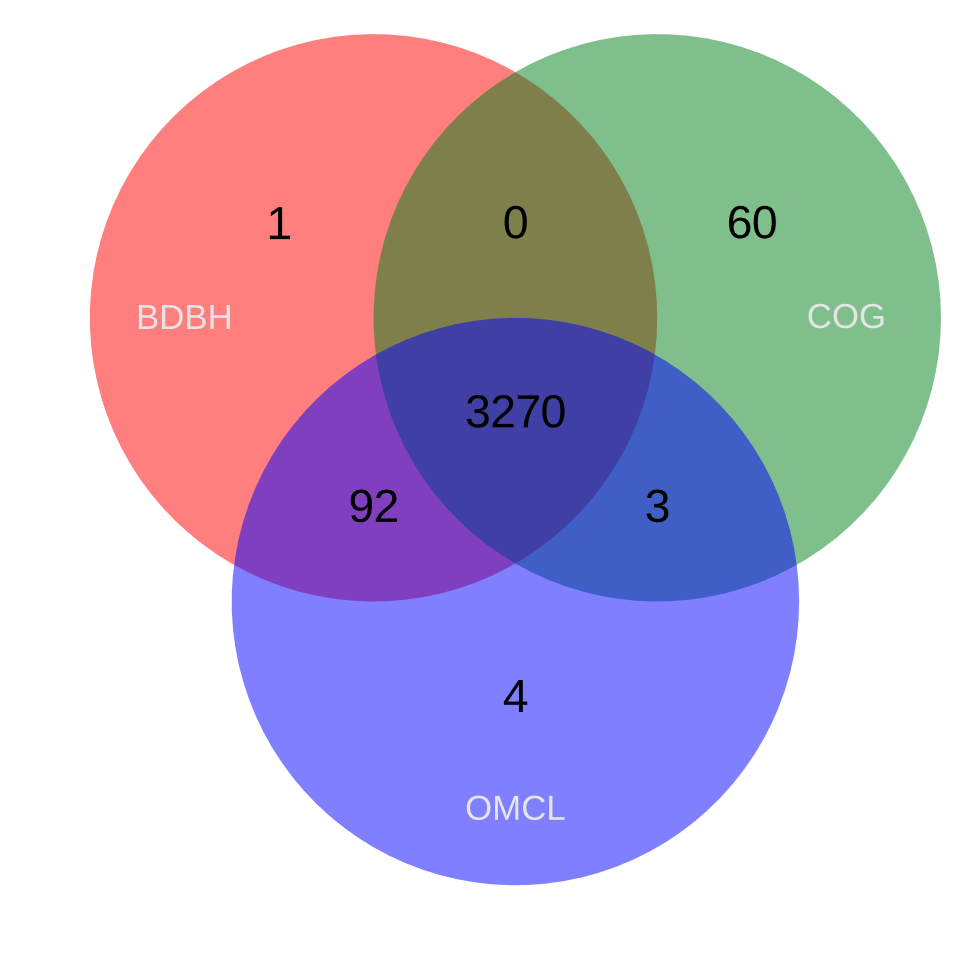
**


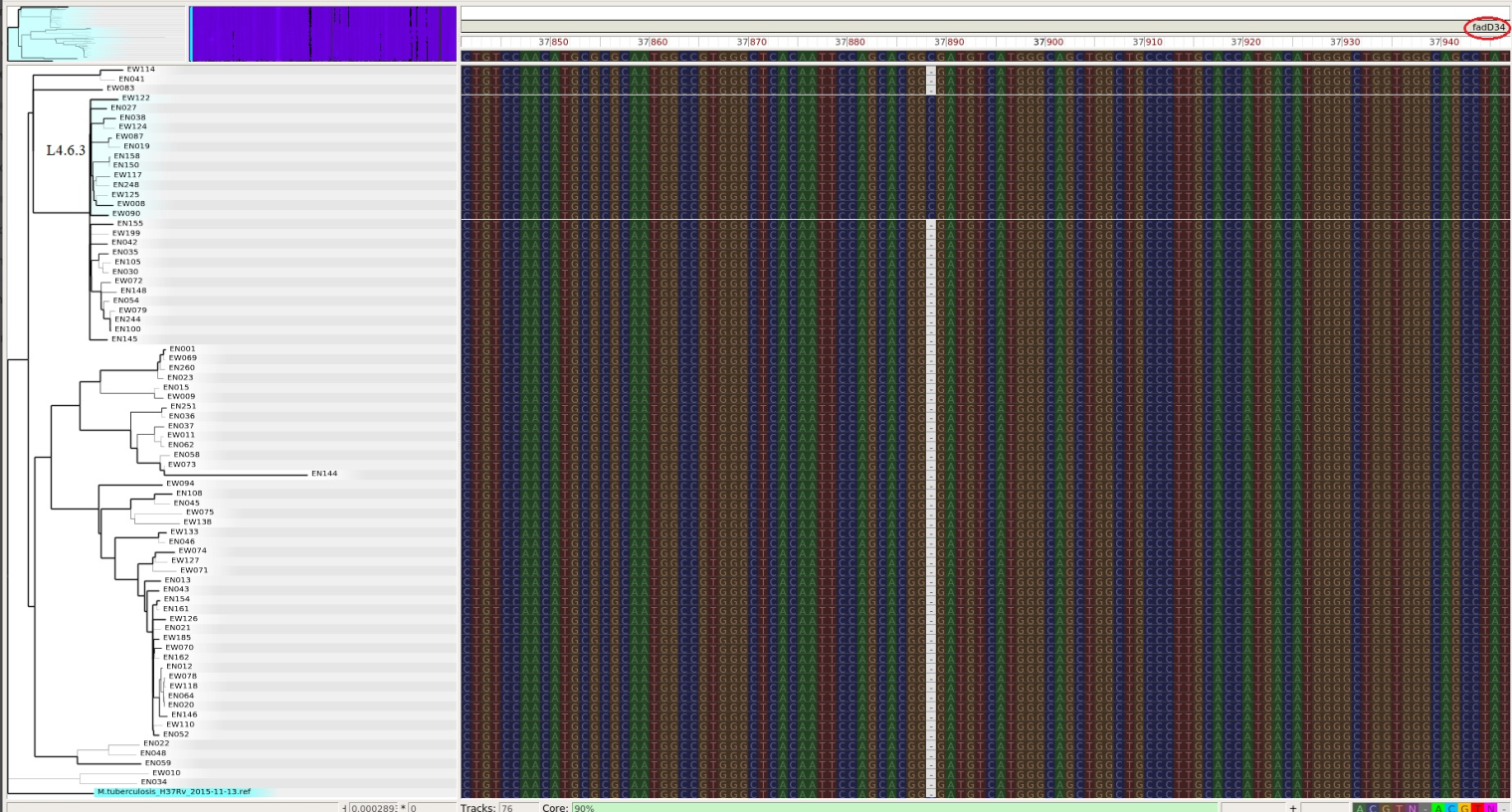
 **S6 Fig.**  **Comparative analysis with parSNP. The left side shows the core phylogeny of 75 isolates using H37Rv as a reference** (**GenBank accession number** [**NC_000962.3**](https://www.ncbi.nlm.nih.gov/nuccore/NC_000962.3)**).** The right side corresponds to a multi-genomic alignment against the phylogenetic tree. The red letter (A) in the white box shows the variant (SNP) in the *fadD34* gene of high prevalence L4.6.3 genomes.


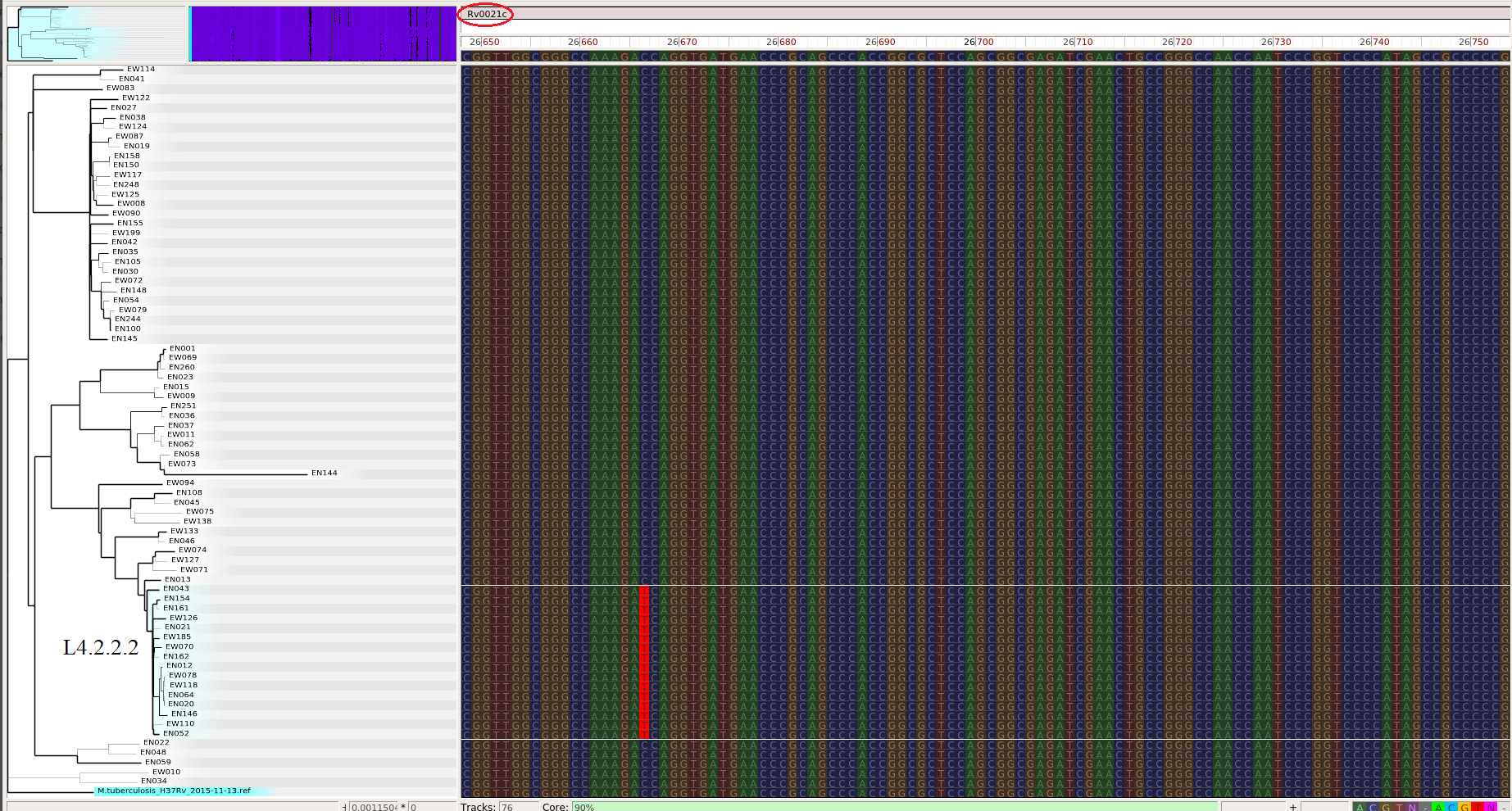


**S7 Fig.** **Comparative analysis with Parsnp.** The left side shows the core phylogeny of 75 isolates using H37Rv as a reference (GenBank accession number [NC_000962.3](https://www.ncbi.nlm.nih.gov/nuccore/NC_000962.3)). The right side corresponds to a multi-genome alignment against the phylogenetic tree. The red letter (A) in the white box shows the variant (SNP) in the *Rv0021c* gene of high prevalence L4.2.2.2 genomes.


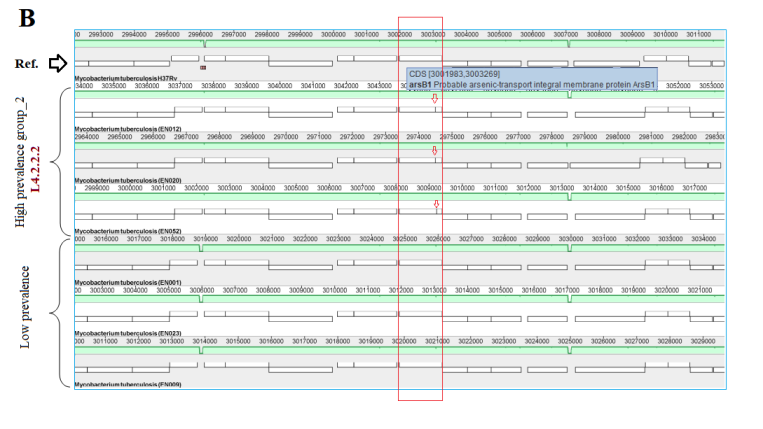

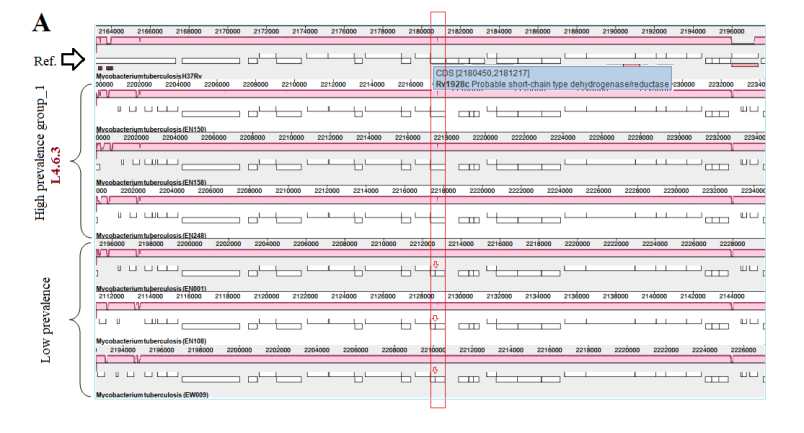
**S8 Fig.** **Comparative analysis with Mauve.** The joined segmented rectangles or squares correspond to the CDS annotated. The blue-shaded rectangle shows the locus annotation for the *Rv1928c* (**A**) and *arsB1* (**B**) genes in H37Rv, reference genome (GenBank accession number [NC_000962.3](https://www.ncbi.nlm.nih.gov/nuccore/NC_000962.3)). (**A**) The red arrow in the red box shows the variant difference of the *Rv1928c* gene between high prevalence L4.6.3 genomes with a complete CDS and low prevalence with two smaller CDS are observed, the first with a premature stop codon due to deletion and the second after the deletion despite having a start codon possibly not be functional. (**B**) The red arrow in the red box shows the genetic variant difference of the *arsB1* gene between low prevalence genomes with a complete CDS and high prevalence L4.2.2.2 with two smaller CDS are observed, the first with a premature stop codon due to deletion and the second after the deletion despite having a start codon possibly not be functional.


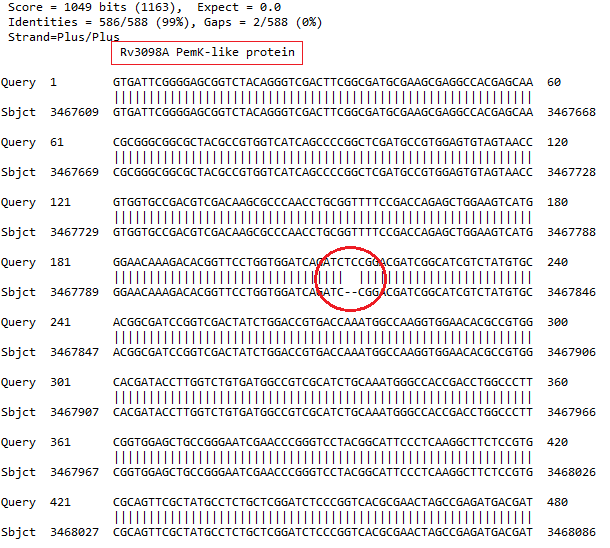

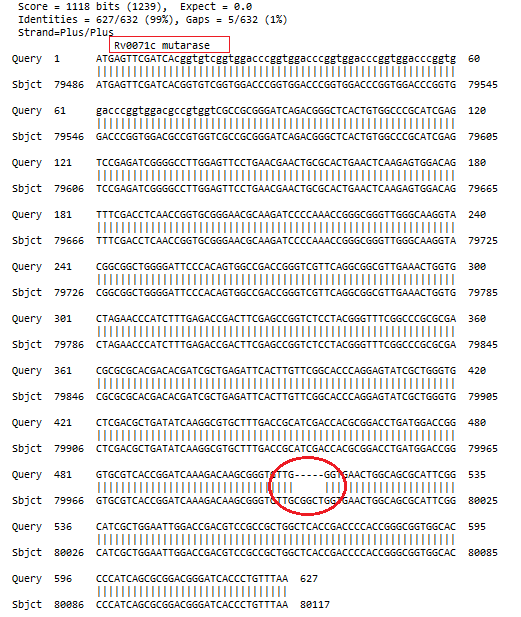


**S9 Fig.** **Comparative analysis by blastn against H37Rv as a reference (GenBank accession number** [**NC_000962.3**](https://www.ncbi.nlm.nih.gov/nuccore/NC_000962.3)**).** The gap in the red circle shows the insertion in the *Rv3098A* and deletion *Rv0071c* genes of high prevalence L4.6.3 genomes.
